# ZmNRT1.1B (ZmNPF6.6) determines nitrogen use efficiency via regulation of nitrate transport and signalling in maize

**DOI:** 10.1111/pbi.14185

**Published:** 2023-10-02

**Authors:** Huairong Cao, Zhi Liu, Jia Guo, Zhongtao Jia, Yandong Shi, Kai Kang, Wushuang Peng, Zhangkui Wang, Limei Chen, Benjamin Neuhaeuser, Yong Wang, Xiangguo Liu, Dongyun Hao, Lixing Yuan

**Affiliations:** ^1^ State Key Laboratory of Nutrient Use and Management, College of Resources and Environmental Sciences, National Academy of Agriculture Green Development China Agricultural University Beijing China; ^2^ Key Laboratory for Agricultural Biotechnology of Jilin Provincial Institute of Agricultural Biotechnology, Jilin Academy of Agricultural Sciences (JAAS) Jilin China; ^3^ State Key Laboratory of Plant Environmental Resilience, College of Biological Sciences, Center for Crop Functional Genomics and Molecular Breeding China Agricultural University Beijing China; ^4^ Department of Nutritional Crop Physiology, Institute of Crop Science University of Hohenheim Stuttgart Germany; ^5^ National Key Laboratory of Wheat Improvement, College of Life Sciences Shandong Agricultural University Tai'an Shandong China

**Keywords:** Nitrate transport, nitrate signalling, nitrogen use efficiency (NUE), ZmNRT1.1B, ZmNLP3.1, grain yield

## Abstract

Nitrate (NO_3_^−^) is crucial for optimal plant growth and development and often limits crop productivity under low availability. In comparison with model plant Arabidopsis, the molecular mechanisms underlying NO_3_^−^ acquisition and utilization remain largely unclear in maize. In particular, only a few genes have been exploited to improve nitrogen use efficiency (NUE). Here, we demonstrated that NO_3_^−^‐inducible *ZmNRT1.1B* (*ZmNPF6.6*) positively regulated NO_3_^−^‐dependent growth and NUE in maize. We showed that the tandem duplicated proteoform ZmNRT1.1C is irrelevant to maize seedling growth under NO_3_^−^ supply; however, the loss of function of *ZmNRT1.1B* significantly weakened plant growth under adequate NO_3_^−^ supply under both hydroponic and field conditions. The ^15^N‐labelled NO_3_^−^ absorption assay indicated that ZmNRT1.1B mediated the high‐affinity NO_3_^−^‐transport and root‐to‐shoot NO_3_^−^ translocation. Transcriptome analysis further showed, upon NO_3_^−^ supply, ZmNRT1.1B promotes cytoplasmic‐to‐nuclear shuttling of ZmNLP3.1 (ZmNLP8), which co‐regulates the expression of genes involved in NO_3_^−^ response, cytokinin biosynthesis and carbon metabolism. Remarkably, overexpression of *ZmNRT1.1B* in modern maize hybrids improved grain yield under N‐limiting fields. Taken together, our study revealed a crucial role of ZmNRT1.1B in high‐affinity NO_3_^−^ transport and signalling and offers valuable genetic resource for breeding N use efficient high‐yield cultivars.

## Introduction

Nitrate (NO_3_^−^) is the predominant form of nitrogen (N) source for nutrition of most terrestrial plants (Crawford and Glass, 1998; Gao *et al*., 2022; Hirel *et al*., 2007). NO_3_^−^ is readily dissolved and lost through leaching in most ecosystems and agricultural soils, leading to highly variable NO_3_^−^ concentration in both time and space (Cameron *et al*., 2013; Gallardo *et al*., 2009; Miller *et al*., 2007). To acclimate to these fluctuations, plants evolved the spectacular physiological and developmental plasticity including modifying root architecture, adjusting NO_3_^−^ uptake and transport, and optimizing NO_3_^−^ metabolism (Giehl and von Wiren, 2014; Jia *et al*., 2022; Jia and von Wiren, 2020; Kiba and Krapp, 2016; Liu and von Wiren, 2022; Nacry *et al*., 2013). In addition to being the nutrition in plants, NO_3_^−^ also acts as a potent signal molecule that modulates genome‐wide gene expression, metabolism, physiology as well as growth and developmental processes (Bouguyon *et al*., 2015; Fredes *et al*., 2019; Poitout *et al*., 2018; Vidal *et al*., 2015). Identifying and genetically manipulating the key components of NO_3_^−^ transport and signalling is crucial for genetic improvement of crop N use efficiency (NUE).

Extensive studies have uncovered a great array of regulators in the NO_3_^−^ signalling in the past two decades (Vidal *et al*., 2020). Among them, NRT1.1‐NIN‐like protein (NLP) signalling cascade is central to the NO_3_^−^ acquisition and signalling. In Arabidopsis and rice, AtNRT1.1 and OsNRT1.1B, respectively, have been shown to mediate the dual‐affinity NO_3_^−^ uptake by roots as well as root‐to‐shoot NO_3_^−^ translocation (Hu *et al*., 2015; Huang *et al*., 1996; Leran *et al*., 2013; Liu *et al*., 1999; Liu and Tsay, 2003; Touraine and Glass, 1997; Tsay *et al*., 1993). As a NO_3_^−^ transceptor, NRT1.1 is also at the centre of a complex network of signalling pathway (Gojon *et al*., 2011; Ho *et al*., 2009; Maghiaoui *et al*., 2020b; Wang *et al*., 2009). In Arabidopsis, upon NO_3_^−^ supply, AtNRT1.1 interacts with the Ca^2+^ channel AtCNGC15 that increases cytoplasmic Ca^2+^. The resulting Ca^2+^ spikes are then decoded by a group of type III calcium‐dependent protein kinases (CPKs), AtCPK10, AtCPK30 and AtCPK32, which in turn phosphorylates master NO_3_^−^ signalling transcription factor AtNLP7 for reprogramming the gene expression and plant development (Liu *et al*., 2017; Riveras *et al*., 2015; Wang *et al*., 2020). Recently, AtNLP7 has been proven to be an intracellular NO_3_^−^ sensor that adjusts metabolic and growth responses (Liu *et al*., 2022). In rice, NO_3_^−^ perception promotes OsNRT1.1B–OsSPX4 interaction and results in ubiquitination and the degradation of OsSPX4 by recruiting an E3 ligase OsNIBP1, which releases OsNLP3 and enables its cytoplasmic‐nuclear shuttling to activate downstream NO_3_^−^ signalling (Hu *et al*., 2019). Several rice NLP members, such as OsNLP1, OsNLP3 and OsNLP4, can cooperatively regulate key genes involved in N uptake and assimilation by directly binding to the NO_3_^−^ response elements (NREs) in their promoters, and play essential roles in improving NUE and grain yield (Alfatih *et al*., 2020; Wang *et al*., 2021; Wu *et al*., 2020; Yu *et al*., 2020; Zhang *et al*., 2022). Their indispensable roles vary on N availability as the relevant function of OsNLP1 and OsNLP3 under N‐sufficient conditions but OsNLP4 under N‐deficient conditions.

A similar function of *AtNRT1.1* and *AtNLP7* orthologous genes is supposed in maize, which is habitat‐adapted to arable soil where NO_3_^−^ is the major available N source. By *in vitro* functional characterization in the heterogeneous expression system *X. laevis oocytes*, ZmNPF6.6 (ZmNRT1.1B) and ZmNPF6.4 (ZmNRT1.1A) have been shown to transport NO_3_^−^ and chloride (Wen *et al*., 2017). *In vitro* assay also shows that ZmNLP5/6/8 activate the NO_3_^−^ response by binding to NRE in the promoters of maize NO_3_^−^‐responsive genes (Cao *et al*., 2017; Ge *et al*., 2020; Wang *et al*., 2018b). However, the *in planta* function of maize NRT1.1 s and NLPs and their contribution to NUE remain to be elucidated.

In this study, we showed the NO_3_^−^‐inducible and plasma‐membrane localized ZmNRT1.1B mediates root NO_3_^−^ uptake and signalling, which in turn modulates downstream N assimilation and plant growth through the regulation of nuclear‐cytoplasmic shuttling of the transcription factor ZmNLP3.1. Loss of function of *ZmNRT1.1B* and *ZmNLP3.1* inhibits growth of maize plants under NO_3_^−^ nutrition. Notably, overexpressing *ZmNRT1.1B* confers significantly higher grain yield under low‐ to moderate‐N supply in the fields. These findings extended our understanding of NO_3_^−^ transport and signalling across plant species and provide valuable gene resources for breeding maize cultivars with improved NUE.

## Results

### The duplicated *ZmNRT1.1B* and *ZmNRT1.1C* diverge in expression pattern and protein localization

**Figure 1:**
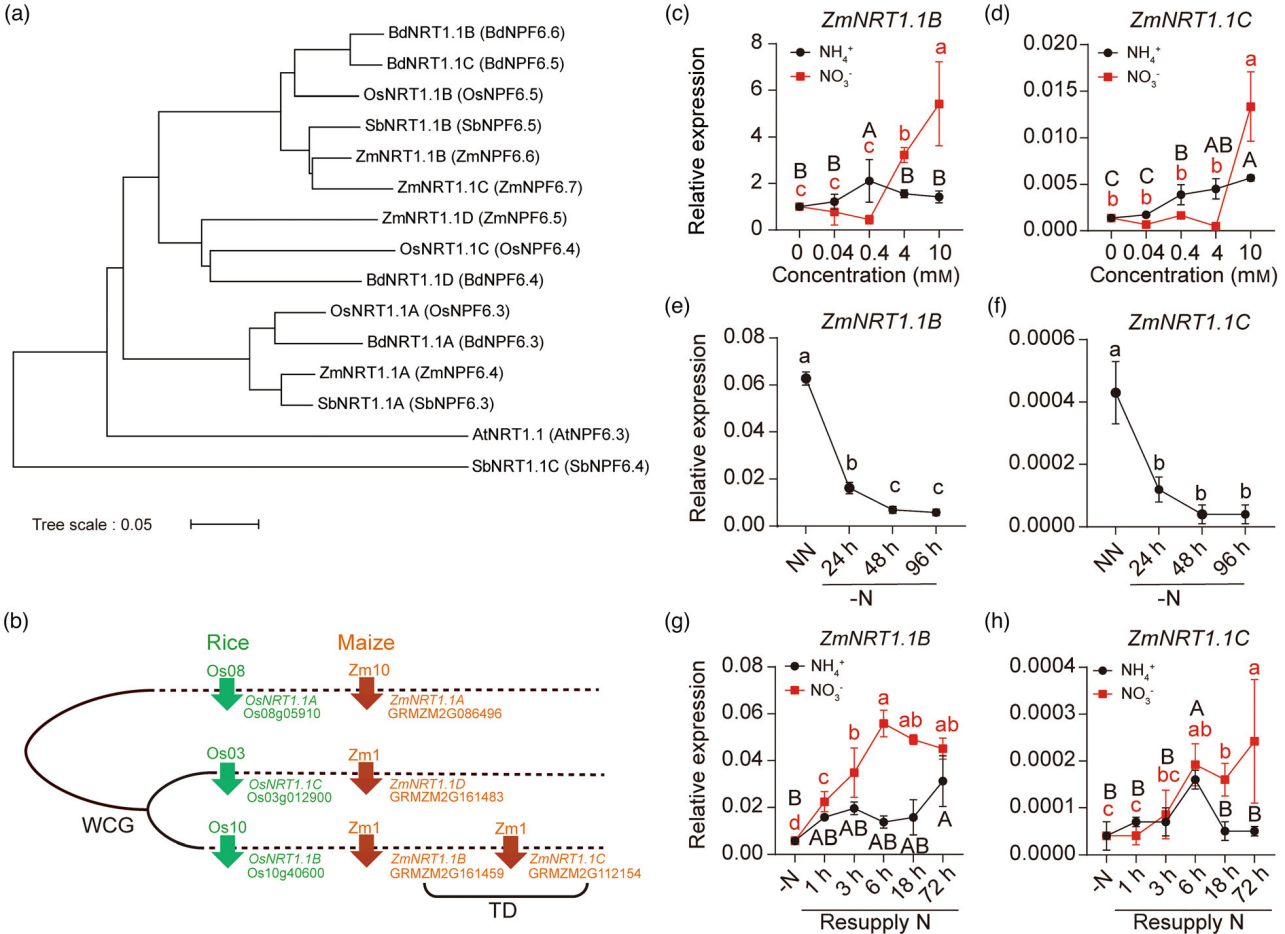
Phylogenetic and collinearity analyses of NRT1.1s and N‐dependent expression pattern of *ZmNRT1.1B* and *ZmNRT1.1C*. (a) Phylogenetic relationship of NRT1.1s in *Zea mays*, *Oryza sativa*, *Sorghum bicolor*, *Brachypodium distachyon* and *Arabidopsis thaliana*. The phylogenetic tree was constructed using MEGA7. (b) Collinearity analysis of NRT1.1 s in maize and rice. WGD, whole‐genome duplication; TD, tandem duplication. (c), (d) Transcriptional responses of *ZmNRT1.1B* (c) and *ZmNRT1.1C* (d) to different N availabilities. (e), (f) Transcript levels of *ZmNRT1.1B* (e) and *ZmNRT1.1C* (f) in response to nitrogen deprivation. Maize seedlings were pre‐cultured with 2 mm NH_4_NO_3_ (NN) for 10 days and then transferred to N‐free nutrient solution (‐N) for 24, 48 or 96 h. (g), (h) Transcript levels of *ZmNRT1.1B* (g) and *ZmNRT1.1C* (h) in response to resupply of nitrate or ammonium for 1, 3, 6, 18 or 72 h of B73 roots. After N starvation for 4 days, maize seedlings were resupplied with either 4 mm KNO_3_ or NH_4_Cl. Bars represent means ± SD (*n* = 3 independent biological replicates). *ZmTUB4* was used as the internal reference. Different letters indicate significant differences at *P* < 0.05 according to one‐way ANOVA followed by Duncan's multiple comparison test.

Gene duplication is a major force of genetic innovation that acquires new genes or novel functions of retaining ones in the organisms (Magadum *et al*., 2013). To gain insight into the genetic evolution of *NRT1.1* genes, we retrieved protein sequences of NRT1.1 s from plant species *Arabidopsis thaliana*, *Zea mays*, *Oryza sativa*, *Sorghum bicolor* and *Brachypodium distachyon*. Comparative genomic analysis showed that whereas only one *NRT1.1* expresses in the Arabidopsis genome, the copy number of *NRT1.1* increases to 3–4 in the monocot species, suggesting that gene expansion of *NRT1.1* occurred after the dicot and monocot specification (Figure 1a). In support, the phylogenetic analysis clustered NRT1.1s into dicot‐ and monocot‐specific clades. Collinearity analysis also showed that during the evolution, *ZmNRT1.1A/OsNRT1.1A* diverged from NRT1.1s, and *ZmNRT1.1B/OsNRT1.1B* and *ZmNRT1.1D/OsNRT1.1C* subsequently evolved through whole‐genome duplication (WGD) (Figure 1b). Interestingly, *ZmNRT1.1B* and *ZmNRT1.1C* encode 89% identical proteins and reside in close vicinity on the chromosome 1, probably deriving from a local tandem gene duplication (Figures 1b and S1).

The evolution of genome‐wide duplicated transporter proteins, for instance in tandem, represents a likely driving force for gene sub‐ or neofunctionalization, either through changing the substrate selectivity, protein localization or spatiotemporal expression patterns of resulting proteoforms (Jia *et al*., 2021; Pottier *et al*., 2022). To gain insights into whether ZmNRT1.1B and ZmNRT1.1C undergo sub‐ or neofunctionalization, we first compared the expression patterns of *ZmNRT1.1B* and *ZmNRT1.1C* in different tissues of field‐grown maize inbred line B73 plants at the silking stage. The quantitative real‐time (qRT)‐PCR analyses showed that *ZmNRT1.1B* exhibited 24–1600 times higher transcript levels than those of *ZmNRT1.1C* in diverse tissues analysed (Figure S2a,b). The *AtNRT1.1* and *OsNRT1.1B* are transcriptionally responsive to NO_3_^−^ addition and crucial for NO_3_^−^ uptake (Hu *et al*., 2015; Huang *et al*., 1996; Liu *et al*., 1999; Touraine and Glass, 1997; Tsay *et al*., 1993). We then investigated the transcriptional responses of *ZmNRT1.1B* and *ZmNRT1.1C* to NO_3_^−^ and ammonium (NH_4_^+^) at various concentrations. Despite not responsive to NH_4_^+^ supply, transcript level of *ZmNRT1.1B* increased steadily with increasing NO_3_^−^ supply (Figure 1c). Distinct to *ZmNRT1.1B*, the transcript level of *ZmNRT1.1C* was increased by both NO_3_^−^ and NH_4_^+^ at high concentrations (Figure 1d).

We then investigated the expression dynamics of *ZmNRT1.1B* and *ZmNRT1.1C* in response to N deprivation and resupply. Whereas N deprivation down‐regulated transcript levels of *ZmNRT1.1B* and *ZmNRT1.1C* (Figure 1e,f), they were significantly up‐regulated by addition of NO_3_^−^ and reached maximum at 6 h (Figure 1g). In contrast to *ZmNRT1.1C*, the expression levels of *ZmNRT1.1B* were not responsive to NH_4_^+^ resupply (Figure 1g,h). These data collectively indicate that the duplicated *ZmNRT1.1B* and *ZmNRT1.1C* diverge in their expression pattern in response to N forms and doses.

We next characterized the subcellular localization of ZmNRT1.1B and ZmNRT1.1C proteins by transiently expressing *ZmNRT1.1B‐eGFP* and *ZmNRT1.1C‐eGFP* fusion in maize mesophyll protoplasts, respectively. The results showed that ZmNRT1.1B‐dependent green fluorescence perfectly co‐localized with the plasma membrane marker FM4‐64, suggesting the plasma membrane localization of ZmNRT1.1B protein (Figure S3). Distinct to ZmNRT1.1B, fluorescence signal of ZmNRT1.1C‐GFP dispersed in the endomembrane‐like structures (Figure S3). These results collectively suggest that the tandemly duplicated ZmNRT1.1B and ZmNRT1.1C differentiate in their expression pattern and protein localization, and these two proteoforms may undergo functional divergence *in planta*.

### Loss of function of ZmNRT1.1B but not ZmNRT1.1C weakens plant growth under replete NO_3_ ^−^ supply

**Figure 2:**
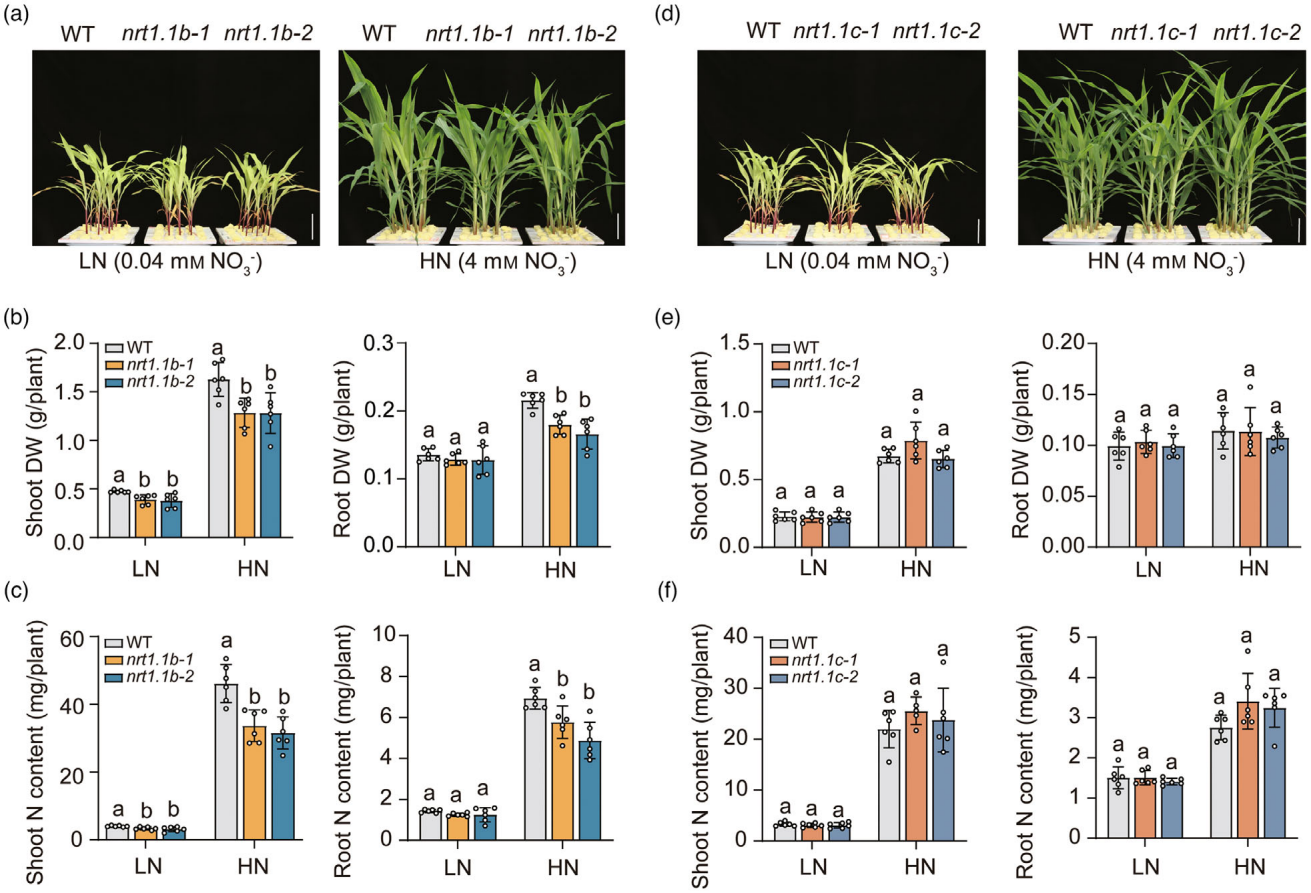
Growth phenotypes of *zmnrt1.1b* and *zmnrt1.1c* mutants under different NO_3_^−^ supply. (a)–(c) Growth performance (a), dry biomass (b) and nitrogen contents (c) of WT and *zmnrt1.1b* mutants under 0.04 mm (LN) or 4 mm KNO_3_ (HN) for 14 days. (d)–(f) Growth performance (d), dry biomass (e) and nitrogen contents (f) of WT and *zmnrt1.1c* mutants under 0.04 mm (LN)or 4 mm KNO_3_(HN) for 14 days. Bars represent mean ± SD (*n* = 6 independent biological replicates). Scale bars, 10 cm. Different letters indicate significant differences at *P* < 0.05 according to one‐way ANOVA followed by Duncan's multiple comparison test.

To assess the physiological significance of ZmNRT1.1B and ZmNRT1.1C, we then generated the loss‐of‐function mutants *zmnrt1.1b* and *zmnrt1.1c* by CRISPR/Cas9‐mediated gene editing in the background of inbred line ND101 (Figure S4a,b). These mutants were hydroponically cultured under low‐ or high‐NO_3_^−^ conditions for 14 days to measure the dry biomass and N content (Figure 2a). In comparison with WT, both *zmnrt1.1b‐1* and *zmnrt1.1b‐2* reduced shoot dry weight by 10%–20% under low and high NO_3_^−^, and root dry weight by 20% under high NO_3_^−^ (Figure 2b). As a consequence, the shoot and root N contents of two *zmnrt1.1b* mutants were significantly lower than those of WT, especially under high NO_3_^−^ (Figure 2c). Relative to WT, none of phenotypic difference was significantly observed in plant growth, biomass and N accumulation of *zmnrt1.1c* mutants irrespective of NO_3_^−^ levels (Figure 2d–f). Collectively, these results indicate that ZmNRT1.1B, but not ZmNRT1.1C, is primarily responsible for NO_3_^−^ uptake and plant growth.

### ZmNRT1.1B modulates high‐affinity NO_3_ ^−^ uptake and signalling *in planta*

**Figure 3:**
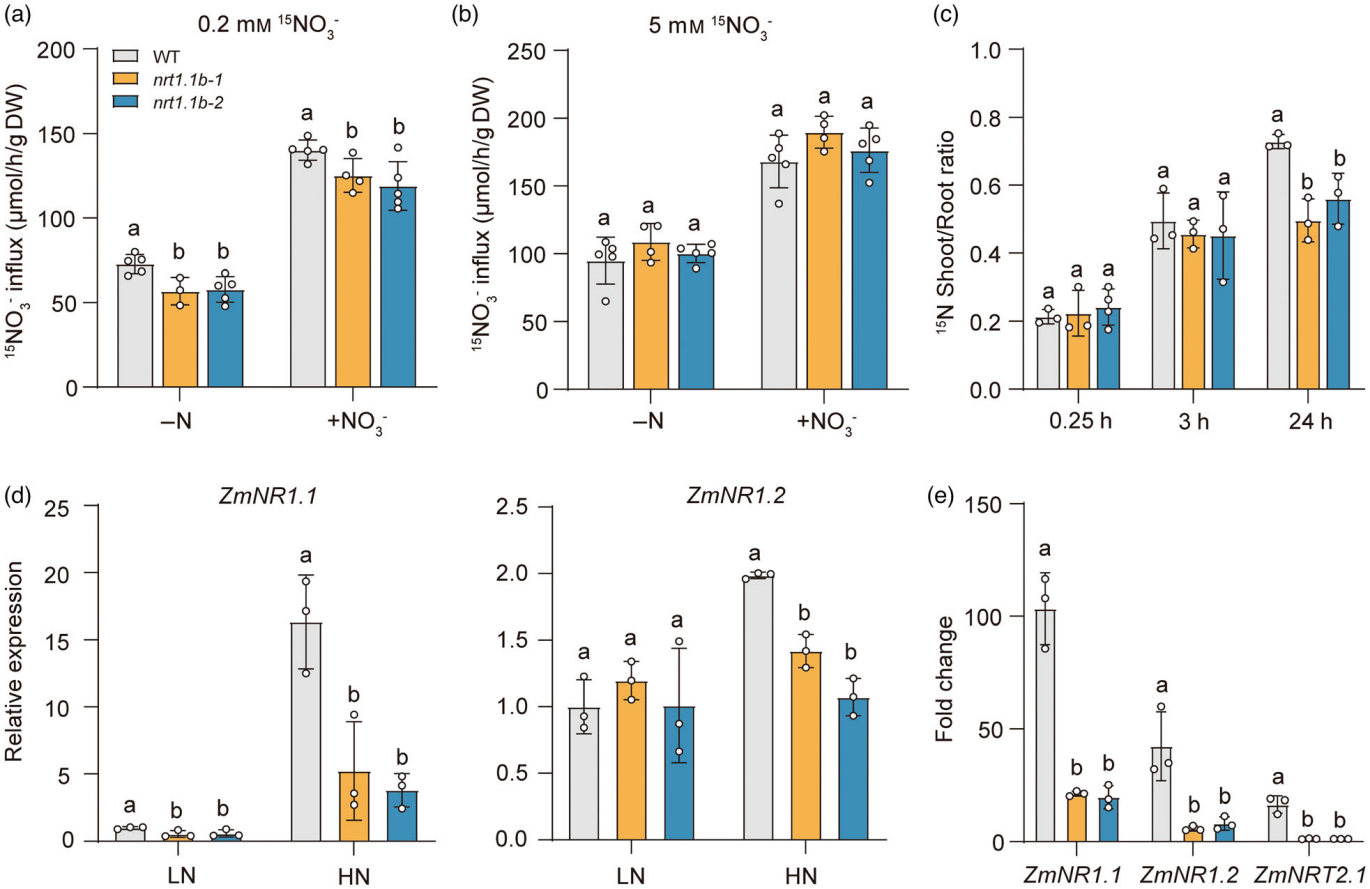
ZmNRT1.1B modulates NO_3_^−^ uptake, root‐to‐shoot translocation and expression of NO_3_^−^‐responsive genes. (a) and (b) Root influx of ^15^N‐labelled NO_3_^−^ supplied at the concentration of 0.2 mm to assess the high‐affinity transport system (HATS) (a) or at 5 mm for the low‐affinity transport system (LATS) (b) of WT and *zmnrt1.1b* mutants. Maize plants were pre‐cultured hydroponically under 2 mm NH_4_NO_3_ for 10 days before transferring to N‐free nutrient solution for 4 days (‐N). N‐deficient plants were then resupplied with 4 mm KNO_3_ (+NO_3_^−^) for 3 h. (c) Nitrate translocation in WT and *zmnrt1.1b* plants. Ratio of shoot/root ^15^NO_3_^−^ contents of different plants in the WT and *zmnrt1.1b* mutants assessed at ^15^N‐nitrate addition for 0.25, 3 or 24 h. (d) Transcript levels of nitrate assimilation genes *ZmNR1.1* and *ZmNR1.2* in WT and *zmnrt1.1b* mutants under 0.04 (LN) or 4 mm KNO_3_ (HN) for 14 days. Relative gene expression of WT grown under low NO_3_^−^ condition was normalized to 1. (e) Nitrate induction levels of *ZmNR1.1*, *ZmNR1.2* and *ZmNRT2.1* transcripts in WT and *zmnrt1.1b* mutants. Ten‐day‐old hydroponically grown maize seedlings were subjected to N starvation for 4 days, and then resupplied with 5 mm NO_3_^−^ for 3 h. Bars represent mean ± SD (*n* ≥ 3 independent biological replicates). Different letters indicate significant differences at *P* < 0.05 according to one‐way ANOVA followed by Duncan's multiple comparison test.

Retarded growth of the *zmnrt1.1b* mutants in the presence of NO_3_^−^ implied that the *zmnrt1.1b* might impair the NO_3_^−^ transport and/or signalling. As ZmNRT1.1B is localized to the plasma membrane, we first characterized the high‐ and low‐affinity NO_3_^−^ uptake by short‐term ^15^N‐labelled NO_3_^−^ influx of hydroponically grown plants (Figure 3a). When plants were pre‐cultured under N starvation for 4 days prior to influx assay, the high‐affinity NO_3_^−^ uptake capacity of *zmnrt1.1b* mutants was ~20% significantly lower than that of WT. For the nitrate‐inducible NO_3_^−^ influx rates, *zmnrt1.1b* mutant plants showed 10%–15% reduction compared with WT after 3 h pretreatment with 4 mm NO_3_^−^. By contrast, no significant difference between WT and mutant plants was detected when they were supplied with 5 mm ^15^NO_3_^−^ to assess the low‐affinity NO_3_^−^ uptake capacity (Figure 3b).

To further determine whether ZmNRT1.1B contributes to the root‐to‐shoot NO_3_^−^ translocation, we quantified the shoot/root ^15^N ratio by extending the ^15^NO_3_^−^ feeding period to 0.25 h, 3 h and 24 h (Figure 3c). Whereas comparable to WT at 0.25 h and 3 h, we observed that shoot/root ^15^N ratio in *zmnrt1.1b* mutants significantly reduced by 31.8% and 23% compared with WT after 24 h, suggesting that ZmNRT1.1B is also crucial for the root‐to‐shoot NO_3_^−^ translocation.

We next examined the expression of N utilization‐related genes in WT and *zmnrt1.1b* mutant plants cultivated under low‐ and high‐NO_3_^−^ regimes. The transcript levels of NO_3_^−^ assimilation genes including *ZmNR1.1* and *ZmNR1.2* consistently and significantly decreased under high NO_3_^−^, suggesting that NO_3_^−^ assimilation may be compromised (Figure 3d). We further assessed the expression of primary NO_3_^−^ response (PNR) marker genes including *ZmNR1.1, ZmNR1.2* and *ZmNRT2.1* to investigate whether *ZmNRT1.1B* is required for NO_3_^−^ signalling. After 3 h short‐term NO_3_^−^ treatments, the transcriptional induction of *ZmNR1.1, ZmNR1.2* and *ZmNRT2.1* in roots of both *zmnrt1.1b‐1* and *zmnrt1.1b‐2* mutants were largely reduced in comparison with WT, suggesting that ZmNRT1.1B is functionally relevant for NO_3_^−^ signalling (Figure 3e). Taken together, these results indicate that ZmNRT1.1B contributes to the high‐affinity NO_3_^−^ uptake, root‐to‐shoot translocation and NO_3_^−^ signalling *in planta*.

### ZmNRT1.1B‐ZmNLP3.1 is required for NO_3_ ^−^ signalling

**Figure 4:**
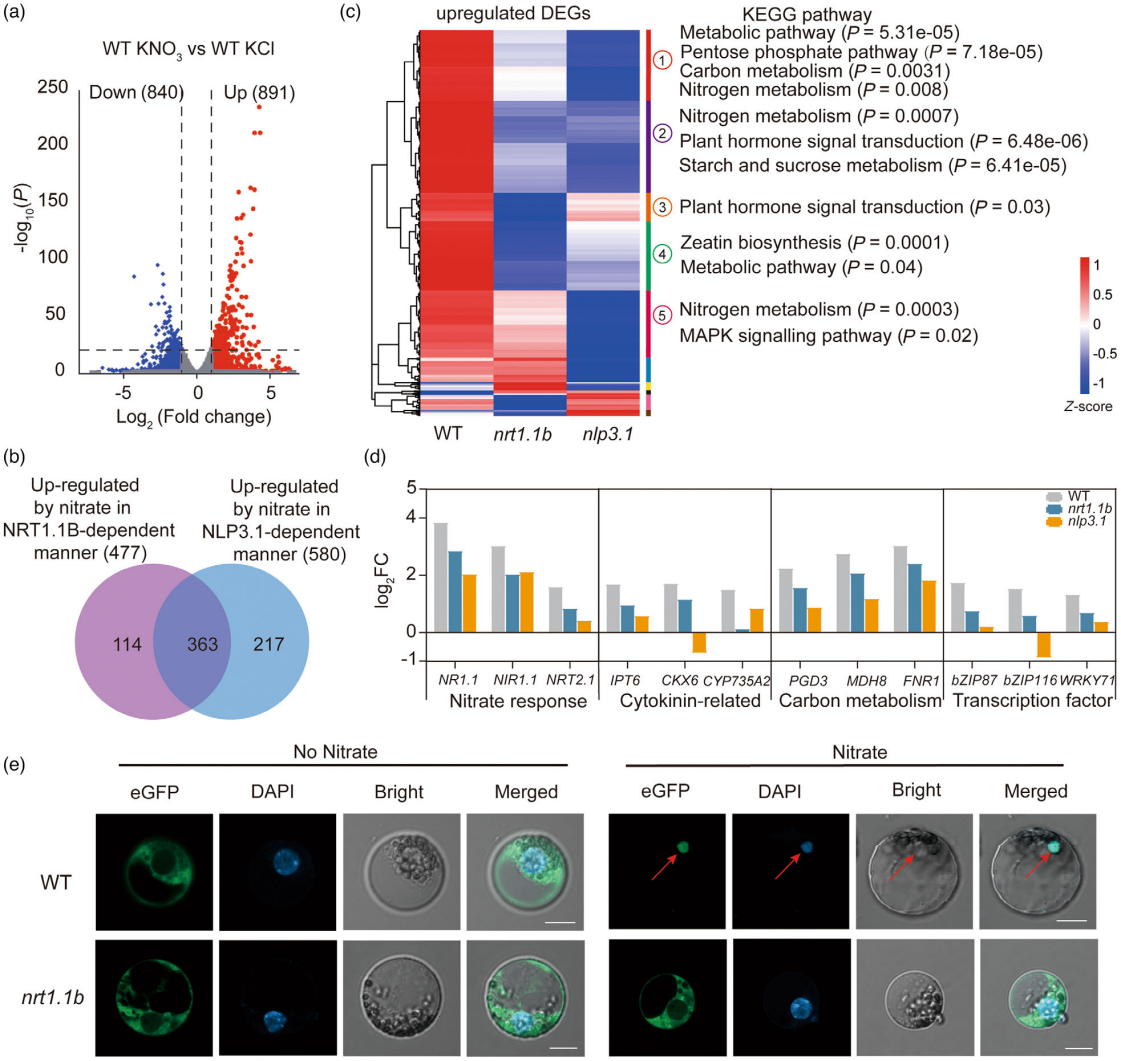
ZmNRT1.1B‐ZmNLP3.1 module regulates NO_3_^−^ signalling in maize. (a) Volcano plots showing the number of differentially expressed genes (DEGs) regulated by NO_3_^−^ in WT. DEGs were clarified with the criteria of adjusted *P* value <0.05 and absolute log_2_FC (fold change) > 1. (b) A Venn diagram showing the overlap gene numbers between ZmRT1.1B‐dependent NO_3_^−^‐up‐regulated genes (477) and ZmNLP3.1‐dependent NO_3_^−^‐up‐regulated genes (580). (c) Hierarchical cluster analysis of the NO_3_^−^‐inducible genes (891 genes) in WT, *zmnrt1.1b* and *zmnlp3.1* mutants, and the KEGG pathway enrichment analysis of respective cluster. The *z*‐score scale bar represents mean‐subtracted regularized log‐transformed FPKM. Full results of KEGG pathway analysis of NO_3_^−^‐up‐regulated DEGs in different clusters are shown in Data S5. (d) NO_3_^−^ treatment regulates the expression of genes mediating NO_3_^−^ response, CK signalling, carbon metabolism and transcription in WT, *zmnrt1.1b* and *zmnlp3.1*. (e) ZmNLP3.1‐eGFP fluorescence expression in WT and *zmnrt1.1b* protoplasts in the presence or absence of NO_3_^−^. Scale bars, 10 μm. Nucleus was visualized with DAPI (blue signal). Images are representative of 10 protoplasts.

In Arabidopsis, AtNRT1.1 and AtNLP7 have been shown to function as the plasma membrane and intracellular NO_3_^−^ sensor, respectively (Ho *et al*., 2009; Liu *et al*., 2022). To investigate the roles of ZmNRT1.1B and ZmNLP3.1 in NO_3_^−^ signalling, we produced two loss‐of‐function mutants of *ZmNLP3.1* via CRISPR/Cas9‐mediated gene editing (Figure S4c). We performed RNA sequencing (RNA‐seq) with the WT, *zmnrt1.1b* and *zmnlp3.1* plants treated with 5 mm KCl or KNO_3_ for 2 h. The RNA‐seq analysis identified 891 NO_3_^−^ up‐regulated and 840 down‐regulated genes in roots of WT (Figure 4a; Data S2). Among these 891 NO_3_^−^‐up‐regulated genes in WT, 477 (53.5%) and 580 (65.1%) genes showed significantly decreased responses to NO_3_^−^ in *zmnrt1.1b* (ZmNRT1.1B‐dependent NO_3_^−^‐up‐regulated genes) and *zmnlp3.1* (ZmNLP3.1‐dependent NO_3_^−^‐up‐regulated genes), respectively (Figure 4b; Data S2–S4). We further observed 363 genes overlapped between ZmNRT1.1B‐dependent NO_3_^−^‐up‐regulated genes (76.1%) and ZmNLP3.1‐dependent NO_3_^−^‐up‐regulated genes (62.6%), and these genes were considered to be co‐regulated by ZmNRT1.1B and ZmNLP3.1 (Figure 4b Data S3).

A hierarchical cluster analysis classified these 891 NO_3_^−^ up‐regulated DEGs into 10 clusters (Figure 4c; Data S5). Among them, the gene set co‐regulated by ZmNRT1.1B and ZmNLP3.1 was assigned into the clusters 1, 2, 3, 4 and 5, KEGG pathway, which were enriched in N metabolism, carbon metabolism, starch and sucrose metabolism, plant hormone signal transduction and MAPK signalling pathways (Figures 4c and S5a; Data S5). Remarkably, genes related to NO_3_^−^ responses, CK signalling and carbon metabolisms showed less induction levels by NO_3_^−^ in *zmnrt1.1b* and *zmnlp3.1* mutants than those in WT (Figure 4d; Data S2–S5). Consistent with the RNA‐seq results, qPCR analysis confirmed that expression levels of NO_3_^−^‐responsive genes *ZmNR1.1*, *ZmNR1.2* and *ZmNRT2.1* were greatly inhibited in *zmnrt1.1b* and *zmnlp3.1* mutants compared with WT (Figure S5d; Data S2–S4). Likewise, of the 840 NO_3_^−^‐repressed genes, ZmNRT1.1B and ZmNLP3.1 were responsible for the expression of 285 (33.9%) and 251 (29.8%) genes, respectively. More than 50% of ZmNRT1.1B‐dependent NO_3_^−^‐repressed genes overlapped with those of ZmNLP3.1 dependence (Figure S5b,c). These data suggest that ZmNRT1.1B and ZmNLP3.1 co‐regulate the expression of NO_3_^−^‐responsive genes through a common signalling pathway.

Given the large proportion of NO_3_^−^‐regulated genes shared by ZmNRT1.1B and ZmNLP3.1, we then explored the genetic relationship of ZmNRT1.1B and ZmNLP3.1 in NO_3_^−^ signalling. We first investigated whether ZmNRT1.1B was sufficient to regulate *ZmNLP3.1* expression. However, we observed that the transcript level of *ZmNLP3.1* was not significantly different between WT and *zmnrt1.1b* mutants under neither low nor high NO_3_^−^ condition (Figure S6). We then tested whether ZmNLP3.1 nuclear retention is regulated by NO_3_^−^ and dependent on function of ZmNRT1.1B. Similar to AtNLP7 in Arabidopsis, we observed that ZmNLP3.1 dispersed in the cytoplasm in the absence of NO_3_^−^, and ZmNLP3.1‐eGFP fluorescence was perfectly obviously co‐localized with the nuclear dye DAPI in the presence of NO_3_^−^, suggesting NO_3_^−^ addition promoted ZmNLP3.1 nucleus retention. Strikingly, the loss of function of ZmNRT1.1B completely prevented ZmNLP3.1 cytoplasmic‐nuclear shuttling (Figure 4e). Taken together, these results indicate that ZmNRT1.1B regulates NO_3_^−^ signalling through modulating cytoplasmic‐nuclear shuttling of ZmNLP3.1.

### ZmNLP3.1 positively regulate NO_3_ ^−^‐mediated plant growth

**Figure 5:**
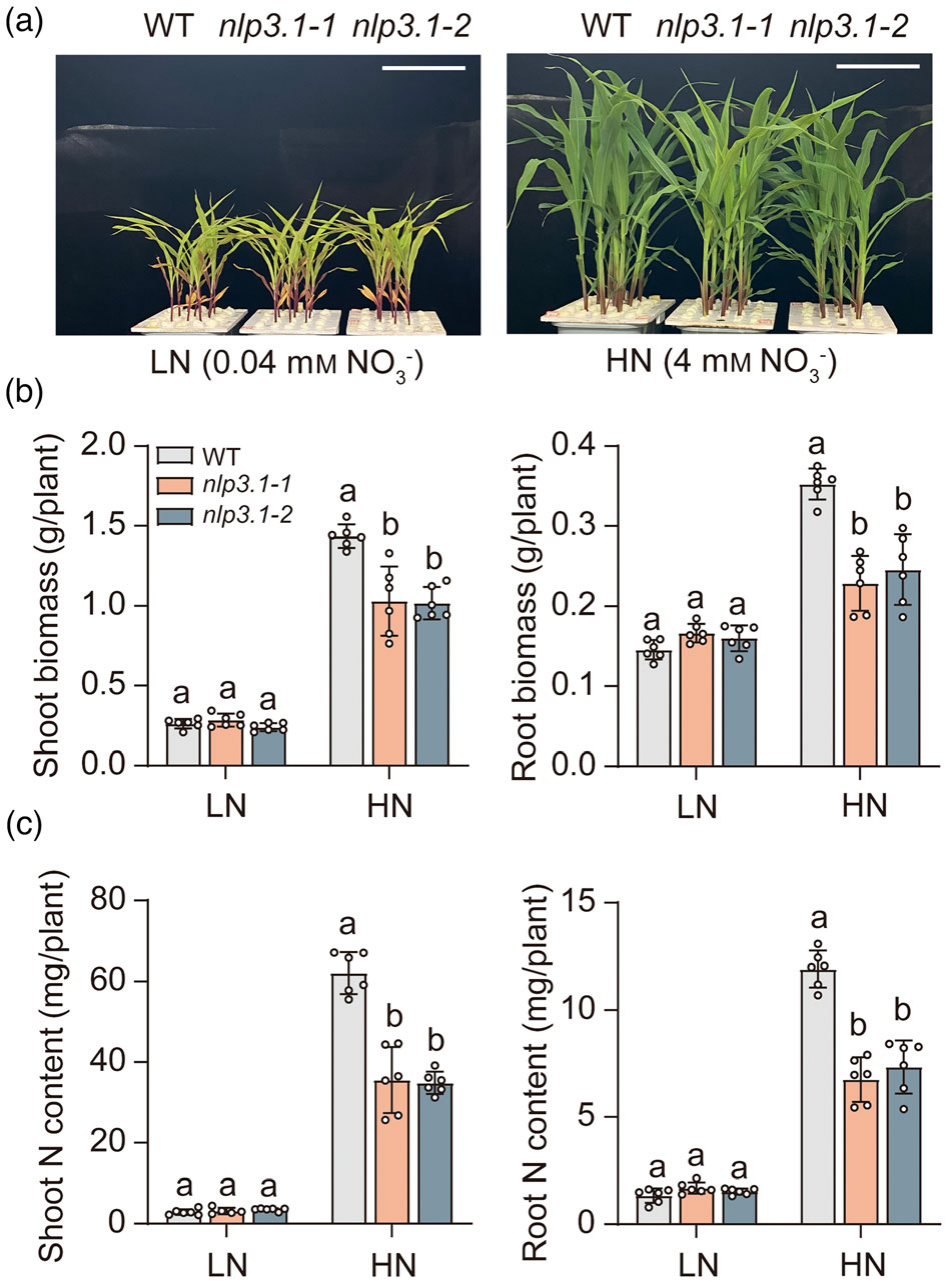
Loss‐of‐function of ZmNLP3.1 weakens plant growth. (a) Growth performance of WT and *zmnlp3.1* mutants under low‐ or high‐NO_3_^−^ conditions. Scale bar, 20 cm. (b), (c) Dry biomass (b) and N contents (c) of WT and *zmnlp3.1* mutants under 0.04 mm (LN) or 4 mm KNO_3_ (HN) for 14 days. Data represent mean ± SD (*n* = 6 independent biological replicates). Different letters indicate significant differences at *P* < 0.05 according to one‐way ANOVA followed by Duncan's multiple comparison test.

To explore the role of ZmNLP3.1 in NO_3_^−^‐regulated plant growth and development, we analysed the growth phenotypes of two *zmnlp3.1* mutants. Although at low NO_3_^−^ supply, growth of WT and *zmnlp3.1* did not differ significantly, the growth promotion by high NO_3_^−^ was obviously suppressed in each *zmnlp3.1* mutant relative to WT (Figure 5a). Under replete NO_3_^−^, the root and shoot dry biomass was significantly reduced by ~35% and ~30%, respectively, compared with WT. Similarly, the total N accumulation in both root and shoot was also decreased by ~40% and ~43%, respectively (Figure 5b,c). We further assessed the transcript levels of NO_3_^−^ uptake and assimilation genes to test whether compromised NO_3_^−^ metabolism and/or acquisition was causal for the weak growth of *zmnlp3.1* mutants. Whereas the transcript level of *ZmNRT2.1* was similar as WT under replete NO_3_^−^ condition, we observed that the expression of both *ZmNR1.1* and *ZmNR1.2* was significantly down‐regulated (Figure S7), suggesting that NO_3_^−^ assimilation is largely compromised, which in turn results in growth defects observed in *zmnlp3.1* mutants.

### The *zmnrt1.1b* and *zmnlp3.1* mutants reduce biomass and yield under field conditions

We tested whether ZmNRT1.1B and ZmNLP3.1 determine plant growth and NUE in the field. In two environments (Beijing and Hainan), the field trials growing *zmnrt1.1b* mutants with sufficient N supply were then performed. Compared with WT plants, *zmnrt1.1b* mutants stably decreased the shoot biomass by ~20%–25% and N contents by ~15%–25%, resulting in a ~30%–40% reduction in grain yield (Figure S8). As a consequence, the NUE of *zmnrt1.1b* mutants reduced by ~44%–50% (Figure S8).

**Figure 6:**
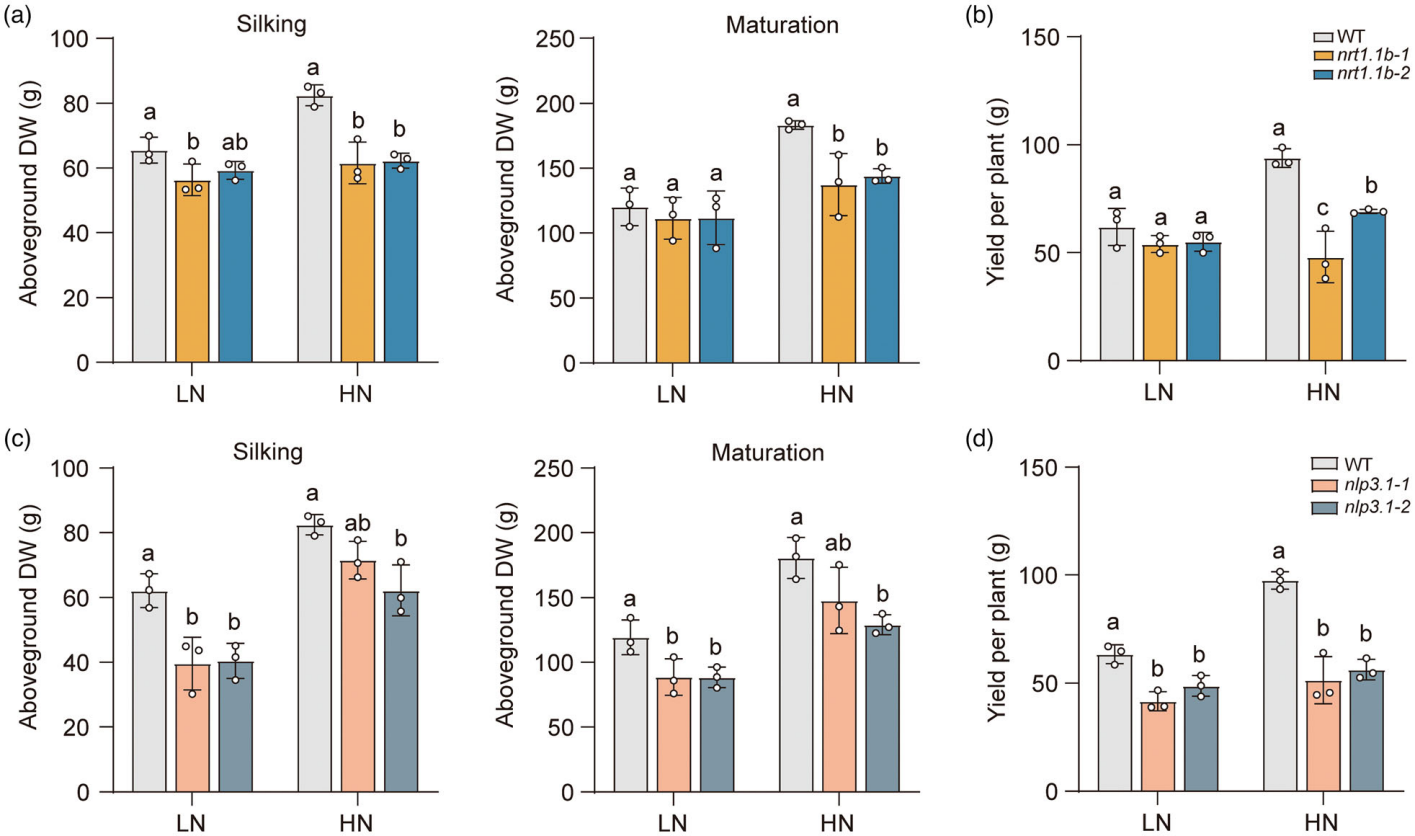
Loss of function of *ZmNRT1.1B* and *ZmNLP3.1* decreases the grain yield in the field. (a), (b) Aboveground biomass at the different developmental stages (a) and yield per plant (b) of the WT and *zmnrt1.1bs* grown under LN or HN conditions. (c), (d) Aboveground biomass at the different developmental stages (c) and yield per plant (d) of the WT and *zmnlp3.1s* grown under LN or HN conditions. The plants were grown in the field plot in Sanya experimental station (Hainan) in 2022. Data represent mean ± SD (*n* = 3 independent biological replicates). Different letters indicate significant differences at *P* < 0.05 according to one‐way ANOVA followed by Duncan's multiple comparison test.

To further evaluate the potential contribution of ZmNRT1.1B and ZmNLP3.1 to N‐dependent yield formation, we planted *zmnrt1.1b* and *zmnlp3.1* mutants under various N regimes. The aboveground biomass of *zmnrt1.1bs* was greatly reduced under high‐N conditions, while no significant difference compared with WT under low‐N during the main growth periods (Figure 6a). For yield formation, *zmnrt1.1b* stably reduced ear length by ~15% and ~12%, resulting in a decrease in yield per plant of ~12% and ~38% compared to WT under low‐ and high‐N conditions, respectively (Figure 6b; Data S6). For *zmnlp3.1* mutants, the aboveground biomass at the silking and maturation stages reduced by 26%–35% and 20%–23%, under low‐ and high‐N, respectively (Figure 6c). Remarkably, *zmnlp3.1* mutants showed significant decreases in ear length (average decrease ~14% and ~8.7%) and ear width (~9% and ~8.7%), resulting in ~29% and ~45% decrease of yield per plant than that of WT under low‐ and high‐N conditions, respectively (Figure 6d; Data S6). These findings indicate that loss of function of *ZmNRT1.1B* and *ZmNLP3.1* results in reduction in plant growth and grain productivity.

### Overexpressing *ZmNRT1.1B* improves grain yield under N‐limiting field

**Figure 7:**
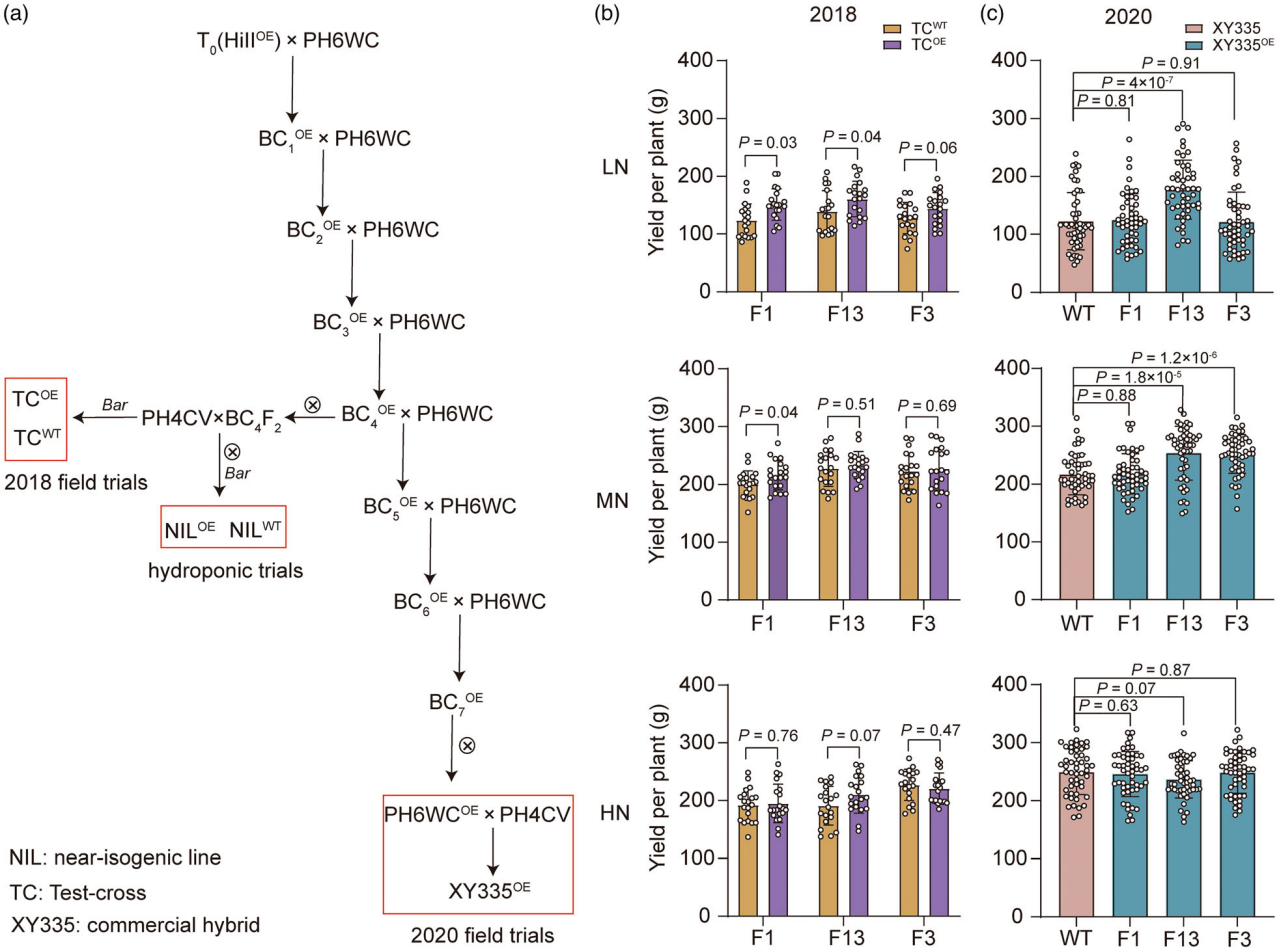
Overexpressing *ZmNRT1.1B* improves grain yield in maize. (a) Genetic construction of transgenic materials used for hydroponic trials, 2018 field trials and 2020 field trials. (b) Yield per plant of TC^WT^ and TC^OE^ lines under low nitrogen (LN), moderate nitrogen (MN) or high nitrogen (HN) condition in the field trial in Changchun (2018). Data represent mean ± SD (*n* = 20 independent biological replicates). TC^OE^ and TC^WT^ represent *ZmNRT1.1B* overexpression lines and corresponding control lines segregating from the same transgenic events. (b) Yield per plant of *ZmNRT1.1B* overexpressed in the XY335 commercial hybrid background under LN, MN or HN condition in the field trial in Changchun (2020). Data represent mean ± SD (*n* = 50 independent biological replicates). Statistical significance was determined by a two‐sided *t*‐test. XY335^OE^ represents homozygous XY335‐overexpressing *ZmNRT1.1B* lines.

To assess the breeding values of ZmNRT1.1B, we created three independent transgenic overexpression lines and introgressed them into the inbred PH6WC, that is female parent of commercial hybrid Xianyu335 (XY335) prevailing in the northeast of China (Figure 7a). We selected two sets of BC_4_F_2_ lines, NIL^WT^ and NIL^OE^. In these NIL^OE^ lines, the transcript levels of *ZmNRT1.1B* were significantly increased up to 10–50 folds higher than those of corresponding NIL^WT^ lines (Figure S9a). All these NIL^OE^ lines exhibited significantly higher tolerance to low NO_3_^−^ availability than NIL^WT^ lines when they were grown in hydroponics, as exemplified by 18%–26% increases in shoot biomass under low NO_3_^−^ (Figure S9b,c).

To further examine whether ZmNRT1.1B could improve grain yield in hybrid breeding practice, we first generated F_1_ testcross, TC^WT^ and TC^OE^, by crossing heterozygous BC_4_F_2_ positive plants with the male parent PH4CV (Figure 7a). We then undertook the field trials in 2018 to evaluate the agronomic traits of the F_1_ plants with overexpressed ZmNRT1.1B (TC^OE^) and the corresponding control (TC^WT^). The plants were grown in the fields fertilized with 0 kg N/ha (0% N, low), 158 kg N/ha (70% N, moderate) and 225 kg N/ha (100% N, high) of urea. Under the low‐N conditions, TC^OE^ plants increased ear length by ~5%–14.2% and hundred‐kernel weight by ~2%–9%, resulting in an ~13%–22.5% increase in yield per plant compared with their WTs (Figure 7b; Data S7). Under the moderate‐N condition, OE1 and OE13 plants increased hundred‐kernel weight by ~3.5%–4.5%, resulting in 2.5%–7% increase of the yield per plant. However, yield improvement in OE1 and OE3 was not observed under the high‐N condition (Figure 7b; Data S7).

We further repeated the back‐crossing (BC_7_) to develop ZmNRT1.1B‐overexpressing inbred lines in PH6WC background, PH6WC ^OE^ (Figure 7a). By crossing them with the male parent PH4CV, the hybrid XY335^OE^ was created. We then undertook the field trials in 2020 by growing the transgenic version of XY335 (XY335^OE^) and non‐transgenic controls (XY335) under various N fertilizer rates (Figure 7c). Overexpression of *ZmNRT1.1B* improved the ear length and hundred‐kernel weight of transgenic XY335 hybrid under both low‐ and moderate‐N conditions (Data S7). Consequently, grain yield per plant of the XY335^OE^ was increased about ~15% and ~11% over XY335 under low‐ and moderate‐N condition, respectively (Figure 7c; Data S7). Under high‐N conditions, these *ZmNRT1.1B*‐OE plants did not show improved yield per plant, hundred‐kernel weight and ear length (Figure 7; Data S7). These results collectively indicate that the enhanced expression of *ZmNRT1.1B* increases yield per se as well as at the hybrid levels, presenting great breeding values for developing N‐efficient maize hybrids.

## Discussion

To adapt to the fluctuant NO_3_^−^ concentration in the environment, plants can activate complex regulatory networks for optimizing N uptake and utilization (Good *et al*., 2004; Li *et al*., 2017; Masclaux‐Daubresse *et al*., 2010; Xu *et al*., 2012). In maize as an essential food and cash crop, however, the molecular mechanism of N acquisition and utilization is poorly understood. In this study, we demonstrated that ZmNRT1.1B mediates the high‐affinity NO_3_^−^ transport and regulates NO_3_^−^ signalling to sustain maize growth and development under both laboratory and field conditions. These findings provide a valuable candidate gene *ZmNRT1.1B* that may innovate strategies to improve NUE in maize.

### Expansion and functional differentiation of *NRT1.1* in grass species

Whole genome‐wide and/or tandem duplications frequently occur in grass species during evolution, leading to functional diversification among the orthologs (Bolot *et al*., 2009). Distinct to Arabidopsis that retains only one *NRT1.1* gene in the genome, the grass species evolved three to four *NRT1.1* members falling into three subclades (Figure 1a; (Plett *et al*., 2010; Wang *et al*., 2020), suggesting that grass duplicated *NRT1.1* may innovate their functions other than NO_3_^−^ transport. In fact, accumulating evidences showed some of the NRT1.1 members retained their functions in the NO_3_^−^transport/signalling; however, some do evolve novel functions (Wang *et al*., 2020). For instance, OsNRT1.1A could regulate flowering time and OsNRT1.1B played an important role in determining the diversity of the root microbiome in rice (Wang *et al*., 2018a; Zhang *et al*., 2019a). Interestingly, in maize, the tandemly duplicated *ZmNRT1.1B* and *ZmNRT1.1C* largely differentiated their expression pattern and protein localization (Figures 1 and S3). For example, expression of *ZmNRT1.1B* was 2–3 orders of magnitude higher than that of *ZmNRT1.1C* (Figures 1c–h and S2). Furthermore, similar to *AtNRT1.1* and *OsNRT1.1B*, *ZmNRT1.1B* is NO_3_^−^‐inducible and plasma membrane bound, while ZmNRT1.1C is localized in the endomembrane system (Figure S3). This suggests that ZmNRT1.1B, but not ZmNRT1.1C, retains the primary function in the NO_3_^−^ uptake, transport and signalling. In support, we observed that the loss of function of *ZmNRT1.1B* causes significant reduction in the plant growth and NO_3_^−^ uptake (Figures 2a–c and 3a). By contrast, the loss of function of *ZmNRT1.1C* resulted in no apparent growth defects under various NO_3_^−^ supply (Figure 2d–f).

Root NO_3_^−^ absorption from soil determines plant growth and productivity for most crops (Dhugga *et al*., 1988). To counteract the changing NO_3_^−^ condition in the environment, plants have evolved two NO_3_^−^ uptake systems: the high‐affinity transport system (HATS) and low‐affinity transport system (LATS) (Crawford and Glass, 1998).

Within the NPF family, AtNRT1.1/OsNRT1.1B was demonstrated as the dual‐affinity NO_3_^−^ transporter (Hu *et al*., 2015; Liu *et al*., 1999). The ZmNRT1.1B functions as the dual‐affinity NO_3_^−^ transporter when it is heterogeneously expressed in *Xenopus oocytes* (Wen *et al*., 2017), while it displayed a high‐affinity NO_3_^−^ transport activity *in planta* (Figure 3a). Together with rapidly responds to exogenous NO_3_^−^ supply in roots at transcript levels, ZmNRT1.1B presumably allows plants to efficiently absorb and utilize NO_3_^−^ according to local N status (Krapp *et al*., 2014). Supporting the efflux activity and preferential mRNA localization of *ZmNRT1.1B* in the central stele, we observed the root‐to‐shoot NO_3_^−^ transport was significantly decreased in the *zmnrt1.1b* plants (Figure 3c; Wen *et al*., 2017). Likewise, the similar function has also been observed for AtNRT1.1 and OsNRT1.1B (Hu *et al*., 2015; Leran *et al*., 2013). These results suggest that AtNRT1.1/OsNRT1.1B/ZmNRT1.1B serves a conserved bidirectional transporter mediating soil NO_3_^−^ uptake and root‐to‐shoot translocation within plants.

### ZmNRT1.1B‐ZmNLPs module is crucial for NO_3_^−^ signalling in maize

NO_3_^−^ additionally functions as a signal molecule that triggers complex transcriptional regulatory networks to efficiently regulate NO_3_^−^ acquisition and plant development (Bouguyon *et al*., 2015; Fredes *et al*., 2019; Poitout *et al*., 2018; Vidal *et al*., 2015). As the first step of NO_3_^−^ signalling, external NO_3_^−^ is perceived by the plasma membrane transceptor NRT1.1, which in turn activates PNR, long‐term transcriptional effects, modification on root system architecture and shoot growth (Ho *et al*., 2009; Hu *et al*., 2019; Jia *et al*., 2022; Jia and von Wiren, 2020; Maghiaoui *et al*., 2020a; Wang *et al*., 2009). Very recently, the intracellular NO_3_^−^ was proven to directly bind to PNR central transcription factor AtNLP7, thereby enabling its conformational change and transcriptional de‐repression (Liu *et al*., 2022). We observed that in the presence of NO_3_^−^, ZmNLP3.1 moved into nucleus. Surprisingly, the loss of function of ZmNRT1.1B completely prevented the ZmNLP3.1 cytoplasm nucleus translocation, even though the underlying molecular mechanism remains unclear (Figure 4e). It is plausible that NRT1.1 leads to the accumulation of cytosolic Ca^2+^ in response to NO_3_^−^ provision, activating CPKs (CPK10, CPK30 and CPK32) that are responsible for phosphorylating NLP7 (Liu *et al*., 2017; Riveras *et al*., 2015; Zhang *et al*., 2019b). As an intracellular NO_3_^−^ sensor, NLP7 acts simultaneously and synergistically with CPK‐dependent phosphorylation for mediating nuclear retention and activating the PNR (Liu *et al*., 2017, 2022). Alternatively, NO_3_^−^ induces the NRT1.1‐mediated degradation of the repression partners of NLP, enabling its activation to promote PNR. In rice, OsNRT1.1B interacted with the phosphate signalling repressor protein OsSPX4, suppressing the nuclear retention of OsNLP3 through protein–protein interaction. The OsNRT1.1B–OsSPX4 interaction was promoted by NO_3_^−^ and degraded by OsNBIP1 (NRT1.1B interacting protein 1), resulting in the release and activation of OsNLP3 (Hu *et al*., 2019). Nevertheless, it remains unknown whether a NRT1.1‐CPK‐NLP‐like module or a NRT1.1‐SPX4‐NLP‐like module exists in maize.

As the NO_3_^−^ sensors, NRT1.1 and NLP7 act at the centre of the NO_3_^−^ signalling network that is crucial for fast genome‐wide transcriptional reprogramming and long‐term developmental changes (Liu *et al*., 2017, 2022; Maghiaoui *et al*., 2020b). We found that more than 40% of the NO_3_^−^‐inducible genes were dependent on the co‐regulation of ZmNRT1.1B and ZmNLP3.1 (Figure 4b,c; Data S2 and S3). Pathway enrichment analysis indicated that ZmNRT1.1B‐ZmNLP3.1 cascade was crucial for N and carbon metabolism, plant hormone signal transduction and MAPK signalling pathways (Figures 4c and S5a; Data S5). Although several studies indicated NO_3_^−^ can induce cytokinin synthesis and transport in roots, few of the molecular mechanisms of N‐dependent cytokinin production are elucidated (Sakakibara, 2021; Takei *et al*., 2004). Here, we showed that ZmNRT1.1B‐ZmNLP3.1 regulate expression of cytokinin biosynthesis genes, such as *ZmIPT6* and *ZmCYPA* (Figure 4d; Data S2–S5). Another significantly enriched functional class was carbon metabolism involving *PGD3*, *MDH8* and *FNR1* (Figure 4d; Data S2–S5). Indeed, as the universal NO_3_^−^‐inducible genes, carbon metabolism genes *AtPGD1*, *AtPGD2*, *AtFNR1* and *AtFNR2* were regulated by the protein kinases CPK10, CPK30 and CPK32 in Arabidopsis (Liu *et al*., 2017). Interestingly, we also observed that ZmNRT1.1B specifically regulated ATP‐binding cassette (ABC) transporters and thiamine metabolism pathways, while ZmNLP3.1 independently regulated biosynthesis of secondary metabolites and phenylalanine, tyrosine and tryptophan biosynthesis pathways (Figure S5a). Furthermore, *zmnrt1.1b* and *zmnlp3.1* exhibited more severe growth defects under high NO_3_^−^ compared with under low NO_3_^−^ conditions similarly with *atnlp2*, *atnlp6* and *atnlp7* (Figures 2a and 5a; Castaings *et al*., 2009; Cheng *et al*., 2023; Durand *et al*., 2023; Konishi *et al*., 2021), suggesting ZmNRT1.1B‐ZmNLP3.1 module may play more important roles under conditions of high NO_3_^−^ availability. Thus, ZmNRT1.1B and ZmNLP3.1 can form a NO_3_^−^ signalling module co‐regulating the expression of NO_3_^−^‐responsive genes; however, they also regulate different downstream pathways.

### Use of ZmNRT1.1B for maize breeding

Crop genetic improvement is the most cost‐effective strategy to tackle the N dilemma for sustainable agriculture towards reducing N demand while pursuing high yield. Among cereals, maize has the highest yield potential that can be realized with more N fertilizer inputs (Wani *et al*., 2021). Our research shows application potential of ZmNRT1.1B for improving the NUE and grain yield in maize breeding. The *zmnrt1.1b* mutants showed growth retardation and yield loss in the field supplied with high N conditions (Figure 6a,b). By contrast, overexpressing of *ZmNRT1.1B* significantly increases ~10% yield of maize test‐cross in the N‐limiting field (Figure 7). In particular, overexpressing *ZmNRT1.1B* in the elite maize hybrid line XY335 performed well in a N‐poor environment and maintained a normal yield even under field with 30% N cut (Figure 7c). Compared with known NO_3_^−^‐transporter genes such as *OsNRT1.1A* and *OsNRT2.3b*, increased expression of *ZmNRT1.1B* did not alter grain yield under high N conditions, which may be related to the high‐affinity N transporter activity (Figures 3a and 7; Fan *et al*., 2016; Wang *et al*., 2018a). Notably, introgression of *ZmNRT1.1B* into the hybrid line XY335 barely impacts the elongation of plant height, minimizing the likelihood of plant lodging that may penalize the yield (Data S7). These suggest that *ZmNRT1.1B* is of great value to crop improvement with an aim of reducing N fertilizer use while maintaining grain yield. In addition, loss‐of‐function *ZmNLP3.1* mutants showed corresponding reductions in biomass production and grain yield (Figure 6c,d). Along with this, field evaluation of breeding value of NO_3_^−^ signalling gene *ZmNLP3.1* will be worthwhile being undertaken in the future.

Taken together, our results demonstrate that a plasma membrane‐localized maize transporter ZmNRT1.1B has great potential for NUE and yield improvement via activating NO_3_^−^ absorption and NO_3_^−^ signalling. When supplied with sufficient NO_3_^−^, ZmNRT1.1B promotes ZmNLP3.1 nuclear retention, which activates the expression of NO_3_^−^ assimilation genes and promotes plant growth. Upon limited NO_3_^−^, ZmNRT1.1B functions as a high‐affinity NO_3_^−^ transporter to acquire NO_3_^−^ and enables higher tolerance of plants to limiting N. Given the significant contribution to NUE, resequencing of *ZmNRT1.1B* and consequent allelic mining in the germplasm pools to uncover relevant natural variants will be valuable in the further study.

## Methods

### Plant materials

Maize (*Zea mays* L.) inbred line B73 was used for gene cloning and expression analyses. The maize loss‐of‐function mutants in the background of ND101 were obtained from the Center for Crop Functional Genomics and Molecular Breeding of China Agricultural University, with the accession number CAUC0713, CAUC0714 and CAUC1783. The construct was transformed into inbred line ND101. The *ZmNRT1.1B* overexpression lines were selected and provided by the Institute of Agricultural Biotechnology, Jilin Academy of Agricultural Sciences. Primers used for isolating CRISPR/CAS9 mutants and overexpression lines are listed in Data S1.

### Growth conditions

For hydroponic experiments, maize seeds were surface sterilized with 10% H_2_O_2_ solution for 30 min, then soaked with saturated CaSO_4_ for 16 h and germinated on filter paper. After seed radicle reached about 1 cm, germinated seeds were vertically placed in a filter paper and immersed in distilled water. Six days after germination, endosperms were removed and seedlings were transferred into the full‐nutrient solution and grown in an artificial climate chamber in a 14 h/28 °C and 10 h/22 °C day‐night rhythm, at a light intensity of 300 μmol/m^2^/s and 60% humidity. The full nutrient solution contained 1 mm K_2_SO_4_, 0.6 mm MgSO_4_·7H_2_O, 0.1 mm KH_2_PO_4_, 0.5 mm CaCl_2_·2H_2_O, 0.1 mm Fe(III)‐EDTA‐Na, 0.5 μm ZnSO_4_·7H_2_O, 0.2 μM CuSO_4_·5H_2_O, 0.07 μm Na_2_MoO_4_·2H_2_O and 1 μm H_3_BO_3_. The N source was supplied in the form of 1 mm (HN_4_)_2_SO_4_ and 2 mm KNO_3_. The pH was adjusted to 5.8.

To assess the transcript levels of *ZmNRT1.1B* and *ZmNRT1.1C* under various concentrations of NH_4_^+^ or NO_3_^−^, seedlings of maize inbred line (B73) were grown in N‐free nutrient solution supplied either with 0.04/0.4/4/10 mm NH_4_Cl or KNO_3_ for 10 days. To investigate the dynamic transcriptional responses to N starvation or resupply, B73 seedlings were hydroponically pre‐cultured under supply of 2 mm NH_4_NO_3_ for 10 days and transferred to N‐free nutrient solution for 24, 48 or 96 h. Afterwards, N was added in the form of 4 mm KNO_3_ or 4 mm NH_4_Cl for 1, 3, 6, 18 or 72 h. Similarly, the NO_3_^−^ induction assay and RNA‐seq analysis were assessed by supplying 5 mm KCl or 5 mm KNO_3_ for 2 h to seedlings starved of N for 4 days after 10‐day pre‐culture under the full‐nutrient solution. Root tissues of maize plants in all experiments were collected for RNA‐seq or qPCR. For phenotypic analysis at the seedling stage, maize seedlings were hydroponically cultivated under the modified Hoagland solution supplied either with 0.04 mm KNO_3_ (LN) or with 4 mm KNO_3_ (HN). Under LN, additional 3.96 mm KCl was provided to maintain the same level of potassium as that in HN. After 14 days, shoots and roots were sampled to assess the biomass accumulation and gene expression.

For organ‐specific expression pattern analysis of *ZmNRT1.1B* and *ZmNRT1.1C*, 0–20 cm underground roots, ear leaf (whole), ear stem (whole), tassels (whole), silk and husk were collected from adult field‐grown B73 plants at silking stage.

### RNA extraction, cDNA preparation and RT‐qPCR

Total RNA was extracted with RNAiso Plus (Takara Biomedical Technology Co., Ltd., Beijing). Approximately 1.5 μg total RNA was cleaned and reversely transcribed into cDNA with gDNA Eraser of PrimeScript™ RT reagent Kit (Takara Biomedical Technology Co., Ltd., Beijing). Then qRT‐PCR assays were performed with TB Green® *Premix Ex Taq*™ II (Takara Biomedical Technology Co., Ltd., Beijing) using CFX96 Real‐Time PCR System (Bio‐rad). The expression of *ZmTUB4* in maize was used for normalizing respective gene expression. The gene‐specific primers used in the qPCR analysis are listed in Data S1.

### RNA‐seq assays

Total RNA was extracted with RNAiso Plus (Takara Biomedical Technology Co., Ltd., Beijing) and RNA integrity was assessed using the RNA Nano 6000 Assay Kit of the Bioanalyzer 2100 system (Agilent Technologies, CA). The mRNA libraries were sequenced on an Illumina Novaseq platform at Novogene (Beijing) and 150 bp paired‐end reads were generated. The reads were mapped to the maize B73 reference genome (B73 RefGen_v4, AGPv4) using HISAT2 (v.2.0.5) with default parameters. Differential gene expression analysis of KNO_3_/KCl was performed using the DESeq2 R package (1.20.0). Corrected *P*‐value ≤0.05 and log2 (fold‐change) ≥1 were set to clarify genes that were significantly differentially expressed. The web server rKOBAS (http://kobas.cbi.pku.edu.cn/) was used to perform KEGG pathway analysis of DEGs in different clusters.

### Plasmid construction and plant transformation

To generate CRISPR/Cas9 knockout lines of *ZmNLP3.1*, two gRNAs targeting the second exon were predicted from CRISPR‐P (http://crispr.hzau.edu.cn/CRISPR2/). Oligo‐F and Oligo‐R were slowly annealed and inserted between two *Bsa*I sites of pBUE411‐2gR. To generate CRISPR/Cas9 knockout lines of *ZmNRT1.1B* and *ZmNRT1.1C*, ZmNRT1.1B‐*pBUE41160* and ZmNRT1.1C‐*pBUE41160* were constructed by the Center for Crop Functional Genomics and Molecular Breeding, CAU. The constructs were transformed into the *Agrobacterium tumefaciens* strain EAH105. The 12‐day‐old immature zygotic embryos of maize inbred line (ND101) were used for *Agrobacterium*‐mediated maize transformation to generate transgenic lines.

To generate transgenic maize lines overexpressing *ZmNRT1.1B*, the open reading frame (ORF) of *ZmNRT1.1B* was amplified using I‐5™ DNA polymerase (Tsingke Biotechnology Co., Ltd., Beijing). The amplified fragment was subsequently cloned into vector pCloneEZ‐Blunt‐Kana/HC cloning kit and sub‐cloned into *p1301* vector through the *BamHI* site. All primes used are listed in Data S1.

### Protein subcellular localization assay

To investigate the subcellular localization of ZmNRT1.1B and ZmNRT1.1C proteins, the coding sequences of *ZmNRT1.1B* and *ZmNRT1.1C* were cloned into *pEZS‐NL* vector. Plasmids were extracted and purified using the Plasmid Midi Kit (Qiagen; No. 12143) following the manufacturer's manual. Leaves of etiolated B73 seedlings grown in dark were digested by Cellulase R10 (Yakult Honsha, Tokyo, Japan) and Macerozyme R10 (Yakult Honsha, Tokyo, Japan) for the preparation of mesophyll cell protoplasts. The empty vector and fusion constructs of ZmNRT1.1B‐eGFP and ZmNRT1.1C‐eGFP were transformed into maize mesophyll cell protoplasts by the polyethylene glycol (PEG)‐induced method. After 12 h incubation in the dark, protoplasts were harvested and eGFP fluorescence was inspected using a ZEISS710 confocal microscope. FM4‐64 was used to counterstain the plasma membrane. Fluorescence signals for eGFP (excitation 488 nm, emission 505–550 nm) and FM4‐64 (excitation 561 nm, emission >575 nm) were detected.

For the analysis of the nuclear‐cytoplasmic shuttling of ZmNLP3.1 by NO_3_^−^ and their dependence on ZmNRT1.1B, the coding region of *ZmNLP3.1* was cloned into *pEZS‐NL* vector and then transformed into mesophyll protoplasts of 13‐day‐old WT and *zmnrt1.1b‐1* mutant. Transformed maize protoplasts were incubated for 12 h in the dark. For nuclear‐cytoplasmic shuttling of ZmNLP3.1, the transformed maize protoplasts were treated with 5 mm KCl or 5 mm KNO_3_ for 2 h. The nuclear dye DAPI (4′,6‐diamidino‐2‐phenylin‐dole) was used to counterstain the nucleus. The GFP fluorescence was then detected using a confocal microscope (ZEISS710, Carl Zeiss).

### N content assays

Maize shoot and root samples were collected from hydroponic and field experiments, respectively. They were then dried at 65 °C for 7 days and ground with ball mill. Approximately 0.3 g material was taken for N concentration determination with a modified Kjeldahl acid‐digestion method (Nelson and Sommers, 1973).

### ^15^NO_3_ ^−^ uptake and translocation assay

Maize seedlings were hydroponically grown under Hoagland nutrient solution supplied with 2 mm NH_4_NO_3_ for 10 days and then subsequently deprived of N for 4 days, followed by resupply of 4 mm NO_3_^−^ for 3 h. The plant roots were then rinsed in 1 mm CaSO_4_ for 1 min, followed by transferring to a solution containing 0.2 mm or 5 mm ^15^NO_3_^−^ (20 atom% ^15^N, Shanghai Research Institute of Chemical Industry) for 6 min ^15^N influx. The roots were finally rinsed in 1 mm CaSO_4_ for 1 min and then separated from the shoots immediately after the final wash in CaSO_4_.

For ^15^N accumulation determination, maize seedlings were hydroponically cultivated inHoagland solution containing 4 mm KNO_3_ for 6 days, and then transferred to a solution containing 4 mm ^15^NO_3_^−^ (20% atom ^15^N, Shanghai Research Institute of Chemical Industry) for 0.25, 3 and 24 h, respectively. The plant roots were then rinsed in 1 mm CaSO_4_ for 1 min. Roots and shoots were dried and then collected immediately. Samples were dried at 65 °C for 7 days. Measurement of ^15^N content was performed by Delta V plus Isotope Mass Spectrometry (DELTA Plus XP, Thermo‐Finnigan, Germany). Uptake activity was calculated as the amount of ^15^N taken up per unit weight of roots per unit time. The ratio between ^15^N in shoot and root was calculated as an indicator of the root‐to‐shoot NO_3_^−^ translocation as described by Hu *et al*. (2015).

### Field trials of maize

Field experiments growing *zmnrt1.1b* mutants were carried out in a completely randomized block design with a single row plot at two different locations in Beijing (N40°13′54″, E116°33′50″) and Sanya (N18°23′23″, E109°11′48″) with largely different climate in 2020. Urea was used as the N fertilizer with approximately 180 kg N/ha. Under aerated field conditions, the supplied urea can be rapid converted to NO_3_^−^ through urease and nitrification, and NO_3_^−^ could be the main available N sources for plants. The NUE defined as the kilogram of grain yield per kilogram of N fertilizer supplied.

For determining the potential contribution of ZmNRT1.1B and ZmNLP3.1 to N‐dependent grain yield, maize plants were grown in two independent field blocks supplied with 0 or 120 kg N/ha. The study was performed in the CAU experimental station (Sanya, Hainan, China) from November 2022 to February –2023. The N was applied in the form of urea and fertilized with a proportion of 40% at the silking stage and 60% at the heading stage. The plants were grown in 32 rows×17 plants for each plot with a 0.5 m distance between rows and replicated three times. Shoots of plants were harvested at silking and maturation stages, respectively. All plants were open‐pollinated, three plants were measured in each row and taken the average of three as a plot replicate.

To evaluate the breeding value of ZmNRT1.1B, large‐scale field tests for ZmNRT1.1B‐OE plants in the Xianyu335 background were performed in the field under three nitrogen conditions: 0 kg N/ha, 158 kg N/ha, 225 kg N/ha from May 2018 to October 2020 at Gongzhuling (N43°31′11″, E124°47′41″). In 2018, plants were grown in 4 rows ×20 plants, and 20 plants were selected to analysis agronomic traits for each genotype. Another field test was also conducted using transgenic plants and Xianyu335 in 2020, with a planting density of 5 rows ×20 plants. For each transformation event and Xianyu335, 50 plants were selected to analyse agronomic traits. The spacing between plants at 2 years was 25 cm (row space) ×30 cm (plant space). For testing yield‐related traits, mature ears were harvested and dried. Yield‐related traits included ear length, ear diameter, hundred‐kernel weight and grain yield per ear.

### Statistical analysis

Data were analysed using software SPSS 20.0 (IBM, Chicago, IL). For pair‐wise comparisons between two groups, the Student's *t*‐test with a two‐tailed distribution was used. For datasets of more than two groups, one‐way ANOVA with Duncan's multiple comparison test was used.

## Accession numbers

The sequences used in this study can be found in the GenBank libraries under the following accession numbers: AtNRT1.1, AT1G12110; OsNRT1.1A, Os08g05910; OsNRT1.1B, Os10g40600; OsNRT1.1C, Os03g01290; ZmNRT1.1A, GRMZM2G086496; ZmNRT1.1B, GRMZM2G161459; ZmNRT1.1C, GRMZM2G112154; ZmNRT1.1D, GRMZM2G161483; ZmNLP3.1, GRMZM2G375675; ZmNR1.1, GRMZM5G878558; ZmNR1.2, GRMZM2G428027; ZmNIR1.1, GRMZM2G079381; ZmNRT2.1, GRMZM2G010280; ZmIPT6, GRMZM2G116878; ZmCKX6, GRMZM2G404443; ZmCYP735A2, GRMZM2G022904; ZmPGD3, GRMZM2G440208; ZmMDH8, GRMZM2G161245; ZmFNR1, GRMZM2G058760; ZmbZIP87, GRMZM2G000842; ZmbZIP116, GRMZM2G132868; ZmWRKY71, GRMZM2G052671. All sequencing data that support the finding of this study have been deposited in the National Center for Biotechnology Information Gene Expression Omnibus (GEO) with the ID: GSE227472.

## Conflict of interest

The authors declare no conflicts of interest.

## Author contributions

H.C., Z.L. and L.Y. conceived and designed the project. H.C. and Z.L. performed most of the experiments. J.G., Y.S., K.K., W.P., X.L. and D.H. participated in some experiments and conducted the field trials. L.C. and J.G. performed the maize transformation. H.C., Z.L., Z.J. and L.Y. analysed the data. Z.W., Y.W. and D.H. advised the research. H.C., Z.J. and L.Y. wrote and revised the manuscript. All authors read and approved the manuscript.

## Supporting information


**Data S1** List of primers used in this study.
**Data S2** List of NO_3_^−^‐inducible (891) and NO_3_^−^‐repressible genes (840) in WT, *zmnrt1.1b* and *zmnlp3.1* mutants.
**Data S3** List of ZmNRT1.1B‐dependent (477) and ZmNLP3.1‐dependent (580) NO_3_^−^‐inducible genes.
**Data S4** List of ZmNRT1.1B‐dependent (285) and ZmNLP3.1‐dependent (251) NO_3_^−^‐repressible genes.
**Data S5** KEGG pathway analysis of NO_3_^−^‐inducible genes (891 genes) in WT among the 10 clusters.
**Data S6** Yield formation of *zmnrrt1.1b* and *zmnlp3.1* mutants grown in the field with two N rates.
**Data S7** Performance of *ZmNRT1.1B* overexpressing lines grown in the field with three N rates.

**Figure S1** Protein sequence alignment of AtNRT1.1, OsNRT1.1s and ZmNRT1.1s.
**Figure S2** Expression pattern of *ZmNRT1.1B* and *ZmNRT1.1C*.
**Figure S3** Subcellular localization of ZmNRT1.1B and ZmNRT1.1C.
**Figure S4** Schematics of mutations CRISPR/Cas9 maize lines of *zmnrt1.1b*, *zmnrt1.1c* or *zmnlp3.1*.
**Figure S5** ZmNRT1.1B‐ZmNLP3.1 regulates expression of NO_3_^−^‐responsive genes.
**Figure S6** ZmNRT1.1B is not required for transcription activation of *ZmNLP3.1*.
**Figure S7** ZmNLP3.1 regulates the expression of NO_3_^−^‐utilization genes.
**Figure S8** The phenotype of the WT and *zmnrt1.1b* mutants in the field.
**Figure S9** Growth phenotypes of *ZmNRT1.1B* overexpression lines under different NO_3_^−^ concentrations.
