# Supplementary material for: ZmNRT1.1B (ZmNPF6.6) determines nitrogen use efficiency via regulation of nitrate transport and signalling in maize
Source: Plant Biotechnol J. 2023 Oct 2;22(2):316–29. doi: 10.1111/pbi.14185 (PMC10826987; doi:10.1111/pbi.14185)
Supplement: Supplementary file 2 — Figure S1 Protein sequence alignment of AtNRT1.1, OsNRT1.1s and ZmNRT1.1s. Figure S2 Expression pattern of ZmNRT1.1B and ZmNRT1.1C. Figure S3 Subcellular localization of ZmNRT1.1B and ZmNRT1.1C. Figure S4 Schematics of mutations CRISPR/Cas9 maize lines of zmnrt1.1b, zmnrt1.1c or zmnlp3.1. Figure S5 ZmNRT1.1B‐ZmNLP3.1 regulates expression of NO3 −‐responsive genes. Figure S6 ZmNRT1.1B is not required for transcription activation of ZmNLP3.1. Figure S7 ZmNLP3.1 regulates the expression of NO3 −‐utilization genes. Figure S8 The phenotype of the WT and zmnrt1.1b mutants in the field. Figure S9 Growth phenotypes of ZmNRT1.1B overexpression lines under different NO3 − concentrations. [file PBI-22-316-s002.docx]

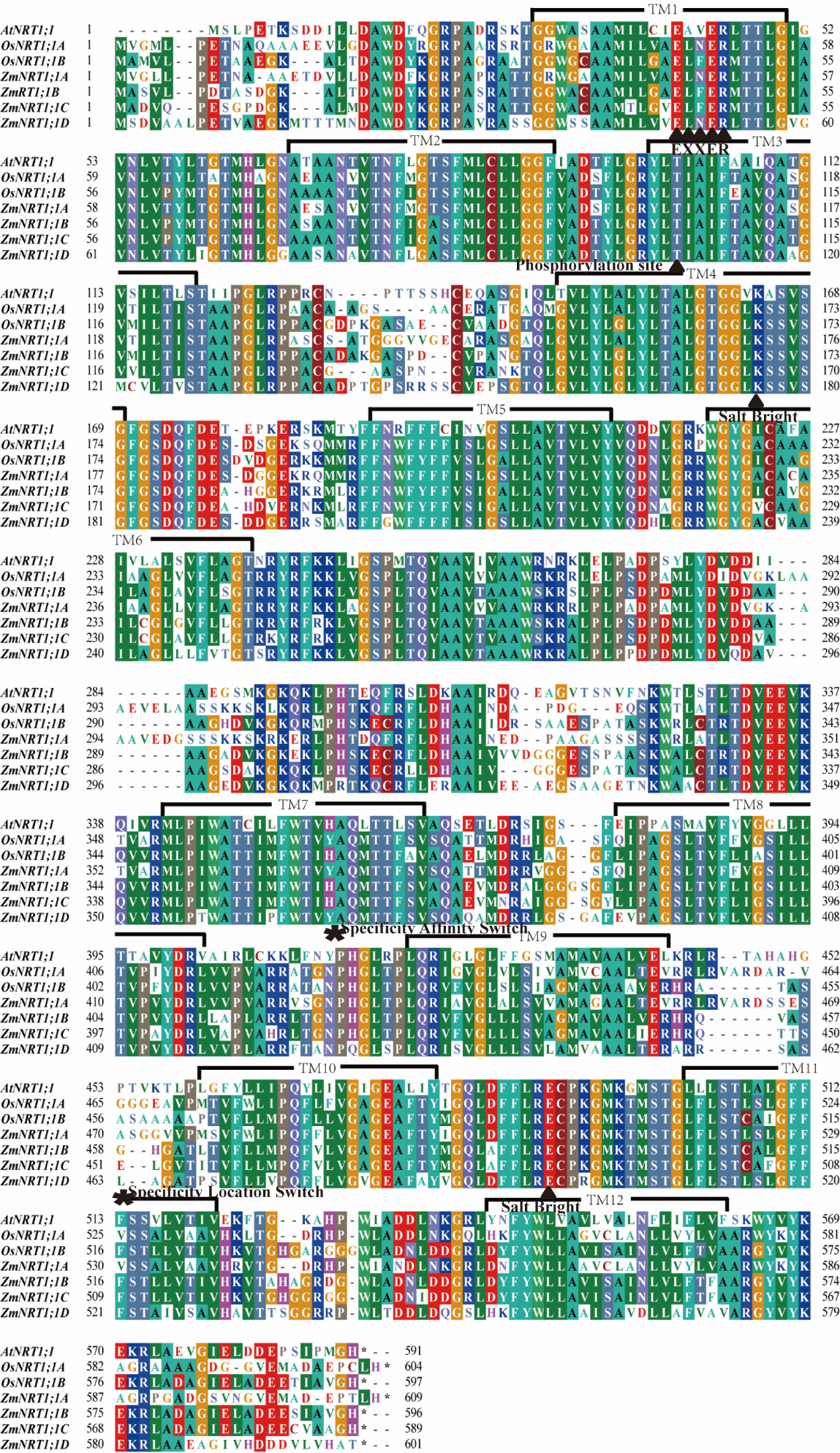


**Figure S1.** Protein sequence alignment of AtNRT1.1, OsNRT1.1s and ZmNRT1.1s. The black lines indicated the conserved transmembrane domain of NRT1.1 protein.


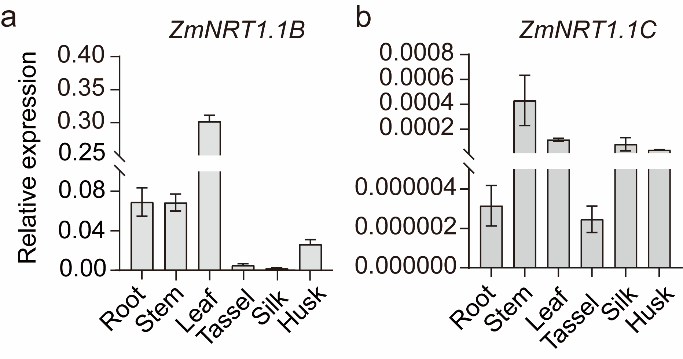


**Figure S2.** Expression pattern of *ZmNRT1.1B* and *ZmNRT1.1C*. Transcript level of *ZmNRT1.1B* (a) and *ZmNRT1.1C* (b) in different organs at the silking stage of WT (B73) plants.


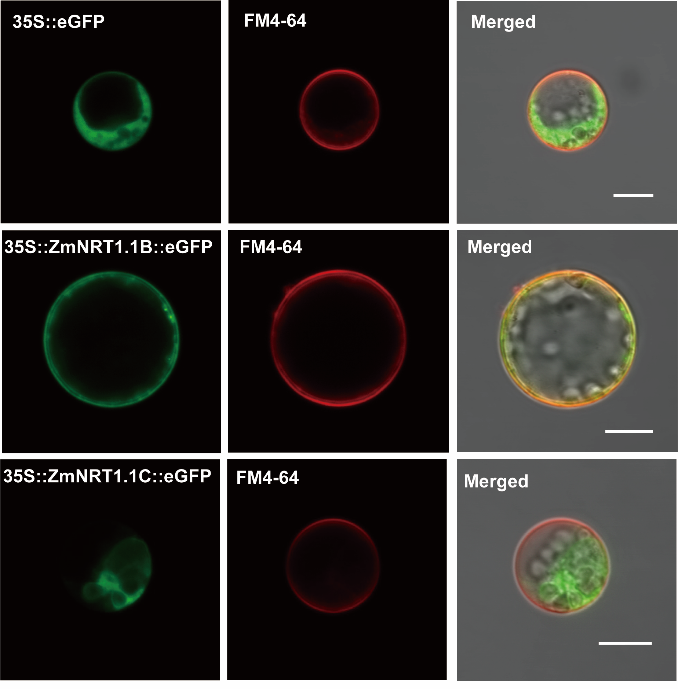


**Figure S3.** Subcellular localization of ZmNRT1.1B and ZmNRT1.1C.

Confocal laser scanning microscopy of 35S::eGFP, 35S::ZmNRT1.1B::eGFP or 35S:: ZmNRT1.1C::eGFP transiently expressed in maize mesophyll protoplast counterstained with membrane marker FM4-64. Scale bar, 10 μm.


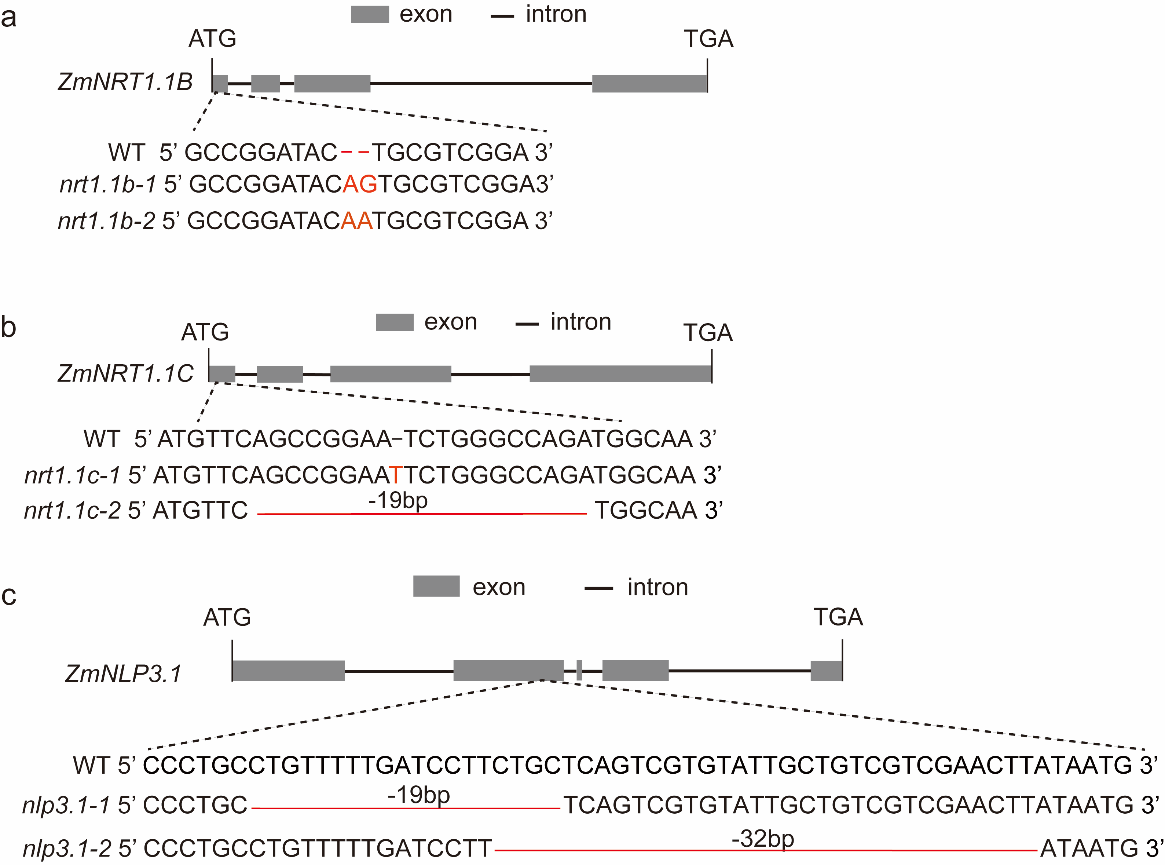


**Figure S4.** Schematics of mutations CRISPR/Cas9 maize lines of *zmnrt1.1b* (a), *zmnrt1.1c* (b) or *zmnlp3.1* (c).


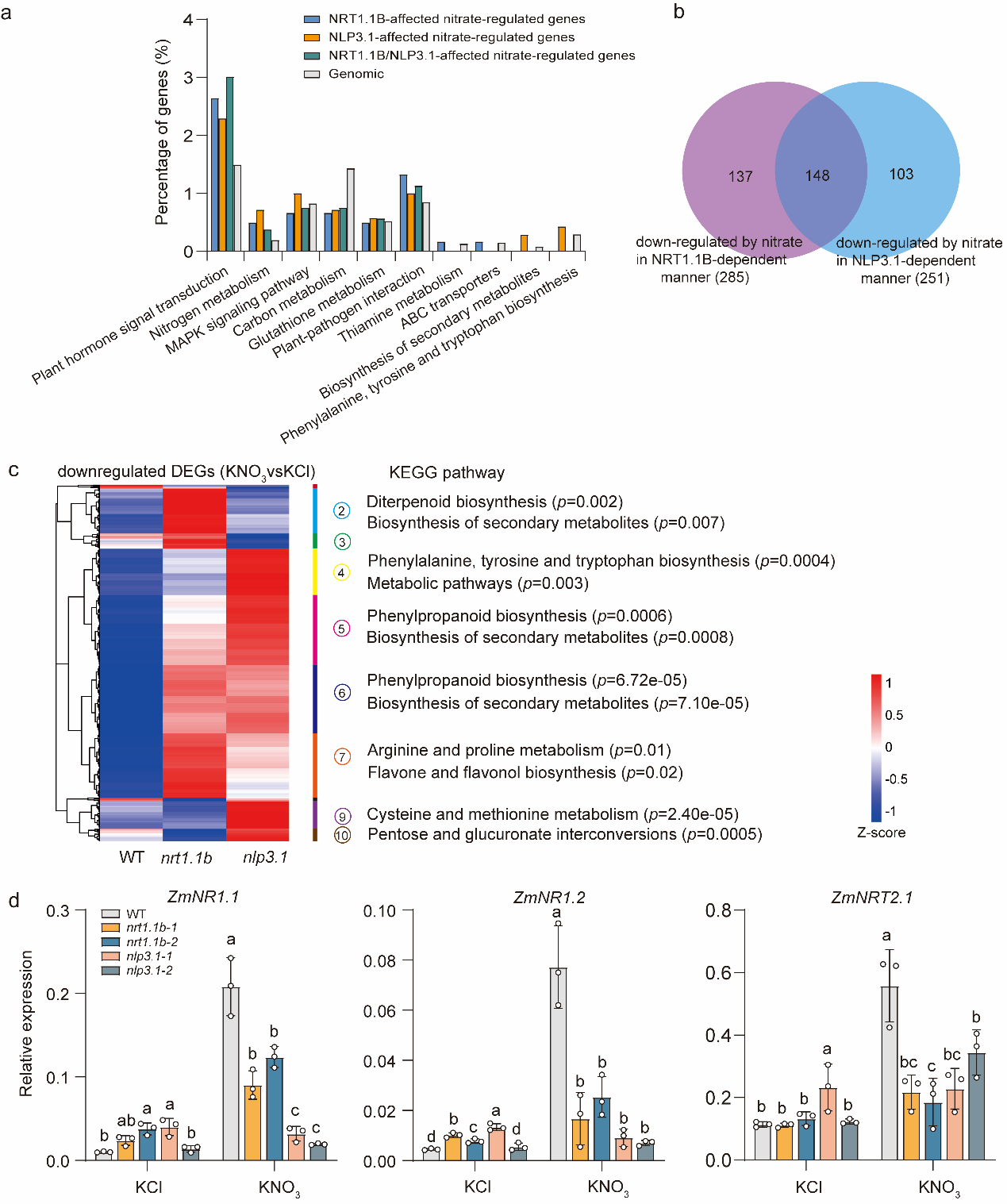


**Figure S5.** ZmNRT1.1B-ZmNLP3.1 regulates expression of NO_3_^-^ responsive genes.

(a) KEGG pathway enrichment analysis of ZmNRT1.1B-dependent, ZmNLP3.1- dependent, or ZmNRT1.1B and NLP3.1 co-regulated NO_3_^-^-responsive genes. Numbers indicate the percentage of genes belonging to each KEGG pathway. Genomic denotes the random genomic control.

(b) A venn diagram showing the overlap gene numbers between ZmRT1.1B-dependent NO_3_^-^-downregulated genes (285) and ZmNLP3.1-dependent NO_3_^-^-downpregulated genes (251).

(c) Hierarchical cluster analysis of the NO_3_^-^-repressible genes (840 genes) in WT, *zmnrt1.1b* and *zmnlp3.1* mutants, and the KEGG pathway enrichment analysis of respective cluster. The z-score scale bar represents mean-subtracted regularized log-transformed FPKM.

(d) The expression of NO_3_^-^-responsive genes *ZmNR1.1*, *ZmNR1.2* and *ZmNRT2.1* in WT, *zmnrt1.1b* and *zmnlp3.1* mutants after NO_3_^-^ treatment. 10-day-old hydroponically grown maize plants were subjected to N starvation for 4 d, and then resupplied with 5 mM KNO_3_ or 5 mM KCl for 2 h. Bars represent means ± SD (n = 3 independent biological replicates). *ZmTUB4* was used as the internal reference. Different letters indicate significant differences at *P < 0.05* according to one-way ANOVA followed by Duncan’s multiple comparison test.


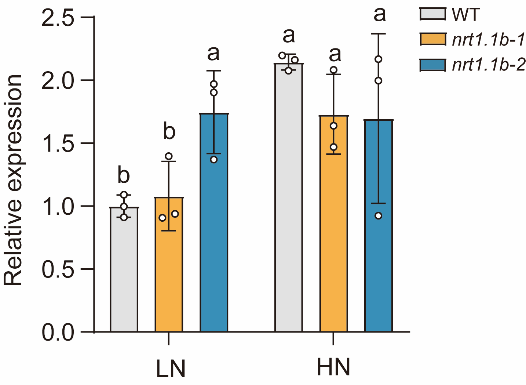


**Figure S6.** ZmNRT1.1B is not required for transcription activation of *ZmNLP3.1*.

Transcript level of *ZmNLP3.1* in WT and *zmnrt1.1b* mutants. Maize seedlings were cultured for 14 d under low (0.04 mM) or high NO_3_^-^ (4 mM) context. Bars represent means ± SD (n = 3 independent biological replicates). *ZmTUB4* was used as the internal reference. Relative gene expression of WT grown under low nitrate condition was normalized to 1. Different letters indicate significant differences at *P < 0.05* according to one-way ANOVA followed by Duncan’s multiple comparison test.


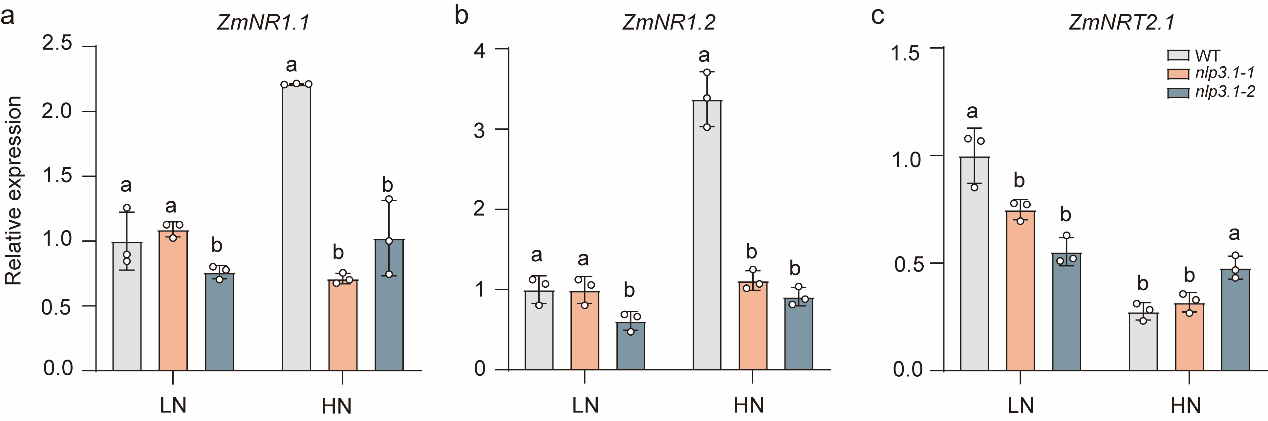


**Figure S7.** ZmNLP3.1 regulates the expression of NO_3_^-^-utilization genes.

Transcript levels of genes mediating NO_3_^-^ assimilation *ZmNR1.1* (a), *ZmNR1.2* (b) and NO_3_^-^ uptake *ZmNRT2.1* (c) in WT and *zmnlp3.1* mutants. Maize seedlings were cultured for 14 d under low (0.04 mM) or high NO_3_^-^ (4 mM) condition. Bars represent means ± SD (n = 3 independent biological replicates). *ZmTUB4* was used as the internal reference. Relative gene expression of WT grown under low nitrate condition was normalized to 1. Different letters indicate significant differences at *P < 0.05* according to one-way ANOVA followed by Duncan’s multiple comparison test.


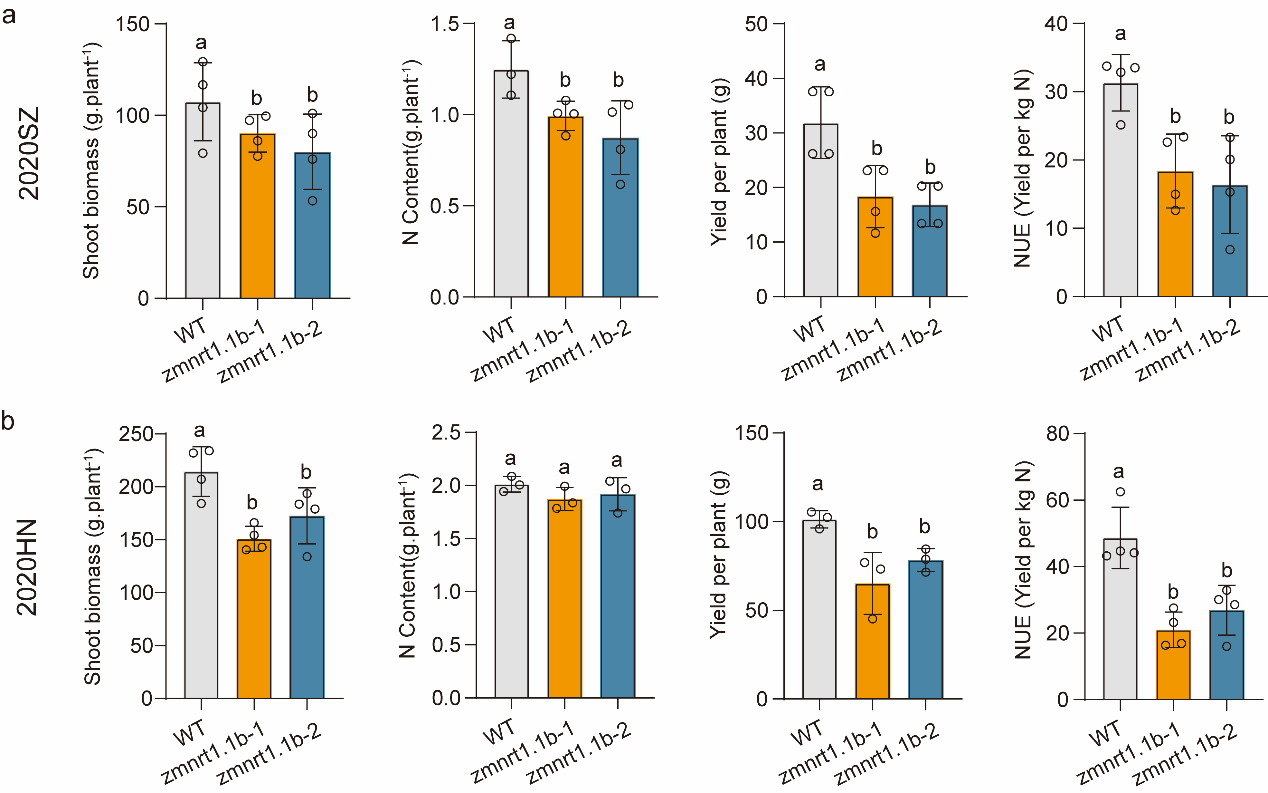


**Figure S8.** The phenotype of the WT and *zmnrt1.1b* mutants in the field.

(a) and (b) Aboveground biomass, N content, yield per plant and NUE of the mature field-grown WT and *zmnrt1.1b* mutants in Shangzhuang (Beijing) (a) and Sanya (Hainan) (b) experimental station in 2020. Data represent mean ± SD (n≥3 independent biological replicates). Different letters indicate significant differences at *P < 0.05* according to one-way ANOVA followed by Duncan’s multiple comparison test.


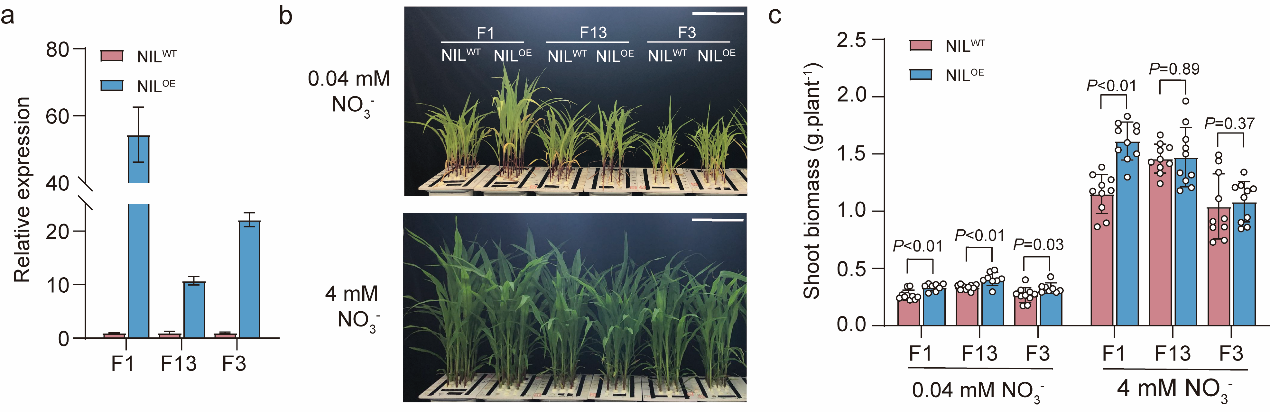


**Figure S9.** Growth phenotypes of *ZmNRT1.1B* overexpression lines under different NO_3_^-^ concentrations.

(a) Transcript levels of *ZmNRT1.1B* in the non-transgenic plants (NIL^WT^) and transgenic plants (NIL^OE^). Bars represent means ± SD (n = 3 independent biological replicates). *ZmTUB4* was used as the internal reference. Relative gene expression was normalized to that of WT.

(b) Growth performance of non-transgenic plants (NIL^WT^) and transgenic plants (NIL^OE^) under low or high NO_3_^-^ condition. Bars, 20 cm.

(c) Aboveground biomass of non-transgenic plants (NIL^WT^) and transgenic plants (NIL^OE^) grown under different N conditions. Bars represent means ± SD (n = 10 independent biological replicates). Maize seedlings were hydroponically grown under supply of 0.04 or 4 mM KNO_3_ for 14 d. Statistical significance was determined by a two-sided t-test.
